# Audit and group feedback in nursing home physician groups: lessons learned from a qualitative study

**DOI:** 10.1186/s12913-025-12355-y

**Published:** 2025-02-11

**Authors:** Gary Y. C. Yeung, Charlotte A. W. Albers, Martin Smalbrugge, Martine C. de Bruijne, Patricia Jepma, Karlijn J. Joling

**Affiliations:** 1 Department of Medicine for Older People, Amsterdam UMC, Location Vrije Universiteit Amsterdam, Boelelaan 1117, Amsterdam, the Netherlands; 2 Aging & Later Life, Amsterdam Public Health, Amsterdam, the Netherlands; 3 Amsterdam Public Health, Methodology, Amsterdam, the Netherlands; 4 Department of Public and Occupational Health, Amsterdam UMC, Location Vrije Universiteit Amsterdam, Boelelaan 1117, Amsterdam, the Netherlands; 5 Amsterdam Public Health, Quality of Care, Amsterdam, the Netherlands

**Keywords:** Quality of health care, Feedback, Quality improvement, Nursing homes, Qualitative research, Peer group, Audit and feedback, Peer group learning, Group feedback

## Abstract

**Background:**

Audit and group feedback (A&F) is an instrument used to encourage healthcare professionals to improve the quality of care. Clinical practice was audited against a set of criteria and fed back to a group by a facilitator. The aim of this study was to gain a better understanding of how physician group feedback sessions in nursing homes were conducted and to what extent they resulted in action planning.

**Methods:**

Fifteen group feedback sessions of the antibiotic A&F program within a nursing home network were audio-recorded, transcribed, and analyzed via the Framework Method for thematic analysis. The coding was performed using the existing Calgary A&F Framework and Cooke’s conceptual model of physician behaviors, and open inductive codes were added.

**Results:**

Elements of the conceptual model and the Calgary A&F Framework occurred within all group feedback sessions. The relationships within the group and with the facilitators were important elements when moving a group from interpreting the results to formulating action plans. Physician groups responded positively to the audit data, particularly if they were among the best performing. The data were met with doubt by physicians who did not recognize their own practice. When exploring potential reasons for lower guideline adherence, groups often considered data quality or external factors such as the choice of non-adherent treatment by locum staff. The degree of reflection on personal factors as explanations for low adherence and the extent to which groups identified learning and improvement opportunities varied: some groups were able to formulate action plans to address data problems and knowledge gaps, whereas others scheduled a follow-up meeting to develop action plans for treatment or prescribing practice changes.

**Conclusions:**

The facilitator was crucial in supporting the group in interpreting the results, steering the conversation towards sharing change cues, and helping the physician group in developing action plans. The degree of reflection and action planning varied by group. By implementing the lessons learned from this study, group feedback sessions can be refined, supporting participants in action planning.

**Supplementary Information:**

The online version contains supplementary material available at 10.1186/s12913-025-12355-y.

## Introduction

Audit & feedback (A&F) is considered an instrument to improve quality in healthcare in which professional performance is systematically reviewed against a set of criteria and fed back to professionals in a structured manner [1]. Previous research has shown small to moderate improvements in desired practices, depending on the measures in the audit and the design of the feedback [2]. The improvements are stronger when baseline performance is low, when feedback is provided by a respected colleague, when it includes clear targets and an action plan for changing practices, and when it is provided regularly in both written reports and verbally [2]. However, the majority of studies have been conducted with primary care physicians in outpatient settings, whereas fewer studies have focused on nursing homes [2, 3]. The extent to which these recommendations apply specifically to the nursing home setting and the specific contextual factors (e.g., nursing home vision, skill mix, and health care service integration) that can influence the impact of improvement interventions remain uncertain [4–7].

A promising approach to A&F delivery involves a facilitator providing feedback to a group of recipients in a group feedback session [8]. Research in the field of (medical) educational feedback indicates that recipients who actively engage with feedback through social interaction are better able to apply feedback to improve their own learning [8–12]. Along with other literature in implementation science and motivational and behavior change theories [8, 10, 13–18], these findings helped Cooke et al. [19, 20] to design and study their A&F projects with physician groups in Canadian hospitals. They developed the Calgary A&F Framework and a conceptual model of physician behaviors in group feedback sessions to better understand why some physician groups were more engaged than others.

The conceptual model of physician behaviors described the recurrent pattern of physician reactions and behaviors during group feedback sessions that was observed each time a finding (i.e., data) was presented: after the initial reaction to the data, physicians tried to establish meaning and creditability to the data before reflections were shared on their own practice, experiences and views on evidence and clinical best practices [19]. Essential in these group discussions were comments (i.e., change cues), highlighting the importance of a performance gap revealed by the data. These change cues usually lead to a discussion on addressing the gap and planning for change (i.e., change talk).

In the Calgary A&F Framework the different A&F design and implementation elements are connected to the outcomes of physician group feedback [20]. Prerequisite activities such as the role and background of the group facilitator, the relationship and interaction between the facilitator and group, the feedback topic, and the visualization and actionability of the feedback were identified as mediating components for physician group feedback sessions. These prerequisite activities were used to better understand how some discussions progress towards action planning, whereas others stall. Cooke’s et al. [19] research showed that interactions between physician group members most often drove discussions to change cues and change talk.

In the Netherlands, approximately twenty nursing homes have been participating with their physician groups in group feedback sessions since 2021 [21]. The group feedback sessions are organized by our research team within the Dutch sentinel nursing home surveillance network, specifically for physician groups in nursing homes as part of an annual A&F program. During the audit period, physician group members record audit forms within their electronic health record on predefined topics, namely appropriate psychotropic prescriptions and appropriate antibiotic prescriptions for lower respiratory tract and urinary tract infections. The research team analyzed the data and a group feedback session was organized with each physician group to discuss their audit results to promote their professional learning and quality improvement activities.

The first cycle of group feedback sessions strongly varied across physician groups. Some sessions were highly interactive, with strong dynamics among participants as well as between facilitators and participants, resulting in concrete improvement plans. By contrast, other groups were more passive, perceiving the session as a presentation and focused on improving the audit rather than the clinical process. In the current study, by examining data from the second cycle, we aim to investigate the reasons for variations to improve the group feedback sessions. Since professional learning via group feedback sessions is relatively new in nursing homes, this study aims to gain a better understanding of how physician group feedback sessions in nursing homes are conducted and result in action planning. This study builds on research and theoretical models developed in other medical domains, notably the Calgary A&F Framework and Cooke’s conceptual model of physician behaviors [19, 20], which are based on a similar setup of the A&F program and our experience with the first cycle. Based on the results of this qualitative empirical study, we formulate lessons learned to improve the group feedback sessions, professional learning and ultimately the quality of care in nursing homes.

## Methods

### Study design

A qualitative approach was chosen to gain better insights into the experiences and behavior of physician groups that participated in the group feedback sessions. Due to the potential for the A&F design and implementation components to influence varying parts of the session in different ways, it was essential to maintain consistency in our analysis to enable a thorough and insightful comparison of the A&F design and implementation components. Consequently, our study focused on the lower respiratory tract infection (LRTI) part of each antibiotic group feedback session (See Supplementary Box 1, Additional File 1). The group feedback sessions were held between April and July 2023.

To guide the analysis, we followed the Framework Method, a form of thematic analysis that is particularly suited for identifying commonalities and differences within qualitative data [22, 23]. It involves analyzing the relationships between the different parts of the data, thereby seeking to derive descriptive and/or explanatory conclusions clustered around themes, which can be derived inductively and deductively.

The consolidated criteria for reporting qualitative research (COREQ) were used as a reporting guideline (Additional File 2), and an audit trail was kept throughout the study to ensure transparency and rigor in the research process.

### Context and setting of the audit and group feedback sessions

Nursing homes in the Netherlands have a unique medical attending position known as the elderly care physician [24]. Elderly care physicians are medical specialists who provide 24/7 on-site medical care to nursing home residents. Depending on the size and medical staff model of the nursing home organization, they work together in physician groups that can also include nurse practitioners, physician assistants, medical residents in elderly care medicine or family medicine, and/or junior doctors. These professionals are all referred to as physicians unless otherwise specified.

In the A&F program, each physician group appoints a contact person who acts as the liaison between the physician group and our research team’s coordinator. The coordinator encouraged physician groups in this cycle to participate in only one of the two A&F topics to enhance focus in the physician groups. Among the 24 physician groups in the nursing home network, nine chose the psychotropic topic, twelve chose the antibiotic topic, and three still chose both (S07, S12, S15). Throughout the audit period, the coordinator maintained regular contact with the physician group’s contact person and invited them to formulate learning objectives with their physician group and specific questions that they wanted the audit to answer. If necessary and feasible, the research team performed additional analyses, in addition to those already conducted for the group feedback session.

In the month before the audit, physicians were invited to attend instruction webinars on the audit procedure (± 30 min). A prerecorded version and written instructions were shared with the physician group’s contact person.

### Group feedback session

Group feedback sessions were planned preferably on-site in coordination with the physician group’s contact person. Physicians obtained continuing medical education credits for their attendance. The group feedback sessions were scheduled to last 60 min, with an additional 30 min for physician groups that participated in both topics (Supplementary Box 1, Additional File 1). The session focused primarily on group discussion. To support this, the feedback information was provided in a PowerPoint presentation (Additional File 3), which was sent to the physician group prior to the group feedback session and used to facilitate the discussion during the group feedback session.

The presentation contained audit results aggregated at the physician group level for adherence to recommendations from the national elderly care physicians’ guidelines [25]. For LRTI, adherence rates were calculated for three recommendations: A) appropriate treatment decisions regarding initiating or withholding antibiotics for LRTI; B) the appropriate use of C-reactive protein (CRP) tests in the diagnosis of LRTI, which was split into two rates, namely percentage of cases that were B1) appropriately tested and B2) appropriately not tested; and C) prescriptions of recommended antibiotics.

The results were displayed in bar charts and compared with the network’s average, the top 10% performing nursing homes within the network (i.e., achievable benchmark [26]) and – when available – the physician group’s previous results. The charts displayed the adherence rate for a specific guideline recommendation alongside the ‘non-adherence’ rate and the ‘unable to assess’ rate. A proportion of the non-adherence was classified as ‘intentionally non-adherent’ if the physician had recorded this in the corresponding field on the audit form. In addition to the charts, the presentation also contained slides with introductory statements to introduce the different audit results and slides with general questions for reflection/discussion (e.g., “What do you notice?”, “Is this what you expected?”).

### Group feedback session facilitators

The sessions were led by a faculty facilitator, together with a researcher as a research facilitator. The faculty facilitator was an elderly care physician with a background as a teacher at our department’s residency program in elderly care medicine. The faculty facilitator’s tasks in the group feedback session were to lead group discussions, support the group in exploring opportunities, barriers, and enablers to improve their practice, and help to formulate potential (learning) objectives and action plans. Before the start of the group session, the faculty facilitators had a two-hour introductory meeting with the research team on the A&F program, the audit measures, and the structure of the group session. In these introductory meetings, some attention was given to didactic skills, and there was some alignment between the facilitators to try to adopt the same approach in every group feedback session. The research facilitator’s main role was to present the slides to the physician group and explain the charts, the data sources, and the data limitations behind the charts.

Three faculty facilitators and four researchers facilitated the group feedback sessions. They were paired based on their availability to participate in the scheduled sessions. Prior to the sessions, the facilitators held pre-meetings to discuss the specific physician group results. A description of all facilitators and research team members and their backgrounds is included in Supplementary Table 1, Additional File 1.

### Ethics and data collection

This study was reviewed by the Medical Ethics Review Committee of Amsterdam UMC, which confirmed that the Medical Research Involving Human Subjects Act (WMO) did not apply to this study (reference number 2023.0261). All group feedback sessions in which the antibiotic A&F topic findings were discussed were completely audio-recorded (*n* = 15). Together with the invitation for the group feedback session, participants were informed about this study. They were provided with the option to object without reason to the audio recording in advance or at the start of the group feedback session. Prior to recording, consent was obtained from all group session participants.

After every group session, both facilitators completed a structured evaluation questionnaire (Additional File 4). This evaluation questionnaire was developed specifically for our study, based on a questionnaire used by general practitioners in an A&F program [27]. Informed consent for the online questionnaires was acquired digitally at the start of the questionnaire.

### Data analysis

Our study followed the seven stages of the Framework Method described by Gale et al. (2013) [22]. GY and CA conducted all stages of the research, in collaboration with other researchers. In the first stage, the audio recordings of the complete feedback sessions were transcribed verbatim by students and checked by a member of the research team by relistening to the complete audio recording. Relistening was also part of the second stage of becoming familiar with and immersed in the data as researchers. In the third stage, four transcripts were coded independently, and after each transcript discrepancies between codes were resolved through discussion to familiarize us with the codebook. The first codes and themes were based on the Calgary A&F Framework and Cooke’s conceptual model of physician behavior and used as a starting point for our codebook to analyze the transcripts (deductive). New (sub-)codes and themes were added inductively to identify actions and recurring patterns that were not sufficiently captured by the deductive codes. After the first four transcripts, the research team was assured that the coding scheme and approach developed were sufficient to apply to the remaining transcripts (stage 4: Developing a working analytical framework). The remaining transcripts were fully coded by one team member, while the other team member checked the coded transcripts and discrepancies were discussed (stage 5: Applying the analytical framework). CA was the initial coder of all group feedback sessions in which GY took part as a research facilitator. In stage 6, the data from each transcript were summarized overall and for each main element of the framework (prerequisite activities, facilitator, physician behavior, and change cues/talk) (charting data into the framework matrix) (Table 2). In the final stage 7, the research team compared the summaries of the different elements of the framework and looked for similarities and differences in terms of how the different sessions progressed through the model. Furthermore, the summaries of all cases were compared to explore different factors of influence on action planning. To triangulate findings, qualitative data in the form of answers to open-ended questions of the evaluation questionnaire filled out by both facilitators after each session were explored for findings that substantiated or deviated from our data.

All qualitative analyses were performed in MAXQDA 2022 (VERBI Software).

## Results

### Participants

Across the fifteen physician groups, 163 physicians participated in group feedback sessions (Table 1). The number of participants per session ranged from three to twenty, reflecting the size of the nursing home organization and physician group that participated. It was also influenced by other factors, such as who was invited from within or outside the physician group (e.g., affiliated pharmacist/physician group, manager), the scheduling of the session, and absence due to medical emergencies.

**Table 1:** Description of the participants and organization of group feedback sessions

Physician group	Total number participants	Size of nursing home organization^a^	Session facilitators^d^		Type of session	Remarks on participants
			Faculty facilitator	Research facilitator		
S01	20	Medium^b^	n/a	FR1	On-site	-Contact person of the group took on the role of faculty facilitator -5 ECPs, 2 APs, 5 medical residents, 8 other participants of affiliated physician group and affiliated pharmacists
S02	20	Large	FF1	FR2	Online	-ECPs, medical residents and junior doctors, exact numbers are unspecified
S03	11	Medium^b^	FF1	FR2	On-site	-7 ECPs, 1 medical resident, 3 junior doctors
S04	8	Medium	FF1	FR2	On-site	-2 ECPs, 4 APs, 2 junior doctors
S05	10	Large	FF2	FR2	Hybrid	-5 ECPs, 1 AP, 1 medical resident, 3 unspecified
S06	4	Medium^c^	FF1	FR3	On-site	-1 ECP, 1 AP, 1 medical resident, 1 junior doctor
S07	13	Largeb	FF3	FR4	On-site	-6 ECPs, 3 APs, 2 medical resident, 1 junior doctor, 1 medical intern
S08	15	Large	FF2	FR2	On-site	-13 ECPs, 2 medical residents, 1 policy advisor
S09	19	Large	FF1	FR1	On-site	-Contact person of the group had the role of both faculty facilitator and research facilitator; FF1 and FR1 attended in a supportive role -11 ECPs, 7 APs, 1 medical resident
S10	5	Medium^c^	FF3	FR4	On-site	-2 ECPs, 2 APs, 1 director of care
S11	3	Medium^c^	FF1	FR3	Online	-2 junior doctors, 1 non-clinical manager of physician group - a medical emergency hindered the ECP and nurse specialist of the location from attending
S12	6	Medium	FF3	FR2	On-site	-4 ECPs, 1 AP, 1 medical resident
S13	8	Medium	FF1	FR1	On-site	-7 ECPs, 1 medical assistant (to take minutes)
S14	8	Medium	FF3	FR3	On-site	-3 ECPs, 3 APs, 1 junior doctor, 1 medical intern
S15	13	Medium	FF2	FR3	On-site	-6 ECPs, 4 APs, 3 junior doctors

^a^Size of nursing home organization: Small = < 150 beds; Medium = < 500 beds, Large = > 500 beds or >15 locations.

^b^Participated in the antibiotic topic with a selection of their locations.^c^Participated with only one location.

^d^For background information on the session facilitators, see Supplementary table 1, Additional File 1.

*ECP* elderly care physicians, *AP* advanced practitioner (nurse practitioners, physician assistants and/or those in training), medical resident in elderly care physician or general practice.

### Calgary A&F Framework and conceptual model of physician behaviors

**Fig. 1:**
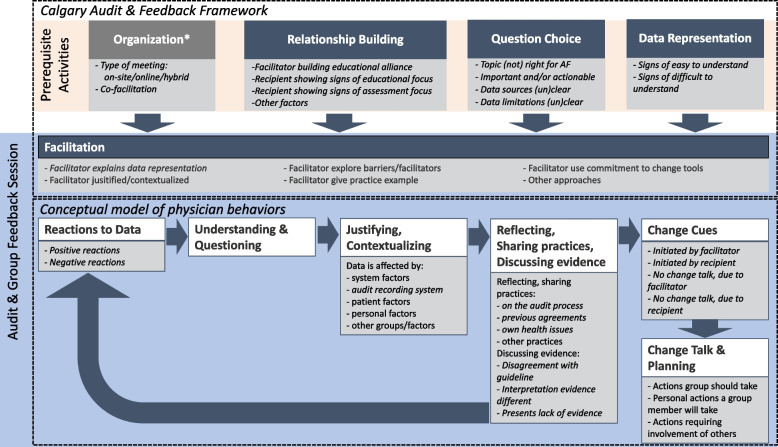
Adapted Calgary Audit and Feedback Framework and conceptual model of physician behaviors The codes used for data analysis in the current study are shown in light gray blocks, with specific codes inductively derived in this study in italic. *New factor included in this study

The results are described following the main components of the adapted Calgary A&F Framework and Cooke’s conceptual model of physician behaviors (see Fig. 1 and Table 2). We included *organization* as an extra component to the existing prerequisite activities, influencing the group feedback session.

**Table 2:** Framework matrix

Group	Prerequisite activities	Facilitator	Physician behavior	Change cues/talk
S01	In-person GFS, in which the discussions were led by an ECP of the PG (co-F1), who was also the network’s CP and part of the research team in the first cycle, having also led GFSs. Prior to the GFS, co-F1 had a meeting with FR1 about the results. The PG also invited the PG of another NH organization, with whom they carry out their pharmacotherapy discussion groups. The other PG had no experience with the audit procedure and not all wards of their own NH organization were included in the audit. co-F1 had also analyzed additional prescription data of both PGs, together with the NH’s pharmacist. Several minutes after the session started, there was a power cut, meaning that the presentation could not be shown on the large screen. Rs had to follow the presentation on laptop screens. R remarked that stratifying results at the department level would be insightful to relate findings to more specific patient populations.	During the presentation, co-F1 also read aloud the official guideline recommendations to the group. co-F1 already contextualized the findings for the group and explicitly mentioned the learning points that she found in the data. co-F1 wanted to discuss action plans at the recap, after all of the LRTI data had been presented. co-F1 ensured that these actions were included in the minutes. For the AB, FR1 mentioned that they were doing well: FR1: (…) No, that’s right, it’s going really well. It’s going better this year than last year. And what's more, that 5%... remains. co-F1: [Name of their organization] is orange. FR1: Yes, [Name of their organization] is indeed orange. And that one exception was someone who was sent to the hospital and, erm, eventually received Augmentin there as well. So.. 95, maybe even 100% are doing fine. (S1) However, there were only nineteen forms: FR1: This works, but it's about nineteen antibiotic prescriptions.. (S1) co-F1: Well, guys, what can be improved? .. CRP? co-F1: What should we write about that? co-F1: Should we do those more often? Or less often? Or…? (S1) When co-F1 asked what could be improved and an R mentioned the “unable to assess” part, co-F1 said that they had already talked about this.	Possible explanations were shared for non-adherence, especially regarding CRP testing. Some Rs disagreed with the guideline. Rs responded openly to critical questions asked by co-F1 about reasons for non-adherence. Regarding AB choice adherence, the PG had an 95% adherence rate with the remaining 5% being intentional non-adherence. R shared that the result was unexpected, “because I see quite a lot of Amoxi passing during the on-call hours anyway” (S1). Rs thought that this might be due to upper respiratory tract infection, cases not captured during the audit, or occurring on wards that did not participate. When Rs heard the reason for intentional non-adherence (prescribed by hospital specialist), they believed that the result should have been 100% adherence. There was a brief discussion about the large proportion “unable to assess” and audit forms not being properly filled out, although little action followed.	Many change cues/talks were not followed by actions, in most cases due to the co-F1 plan to formulate actions at the recap. Regarding the CRP testing, one suggestion was to acquire a second CRP POCT device. The R working on the PG’s budget plan for next year indicated that he could add this to their budget plan. Other actions included adding a guideline-recommended AB to their local formulary that was missing and ensuring that everyone can use the CRP POCT device.
S02	Online GFS with a large PG of an NH with many locations. FF1 already knew some ECPs in the PG.	FF1 mentioned at the beginning that there is little time to discuss everything and repeated this during the session. Time pressure was mentioned as a reason to move on to the next section. The two Fs used different methods to increase the interactivity of the session, which included asking R to physically raise their hands to see if they agreed/disagreed with the introductory statements, keeping long silences, or remarks at a meta-communication level. FR2: [..] we chose nitrofurantoin once. For, yes, a work diagnosis of lower respiratory tract infection, which is also interesting. I don't know if other people ... here ... see any other interesting things or [4-sec silence] recognize? [6-sec silence] For… is this how you expected it to be? [14-sec silence] FF1: I, I think so, listening to this… I’m not hearing any responses, so… Are you surprised by these numbers? (S2) FF1 not only summarized at the end of the LRTI part but also several times during the various discussions and also openly confirmed the accuracy of the summaries with the PG. In the recap, FF1 asked about areas for improvement but received few responses.	Minimal (plenary) interaction in the group, especially during the AB results. Most interaction was on the CRP testing. Rs who joined the online session collectively at a location seemed to interact with each other, although their input was not always heard by the rest. The conversation was also affected by Rs having problems in switching the mic on/off. R noted that adherence rates would look better if the category “unable to assess” was omitted. R indicated as a reason that there is high staff turnover in the PG, including during the audit period, and as a new staff member had not received any instructions on the audit procedure. Rs seem to experience different clinical problems depending on the location in which they practice. For example, regarding CRP POCT, some encountered the absence of qualified staff to perform the test when needed, while others doubted its usefulness for their specific patient population (post-operative rehabilitation patients).	Different issues were raised by Rs, including a lack of knowledge on the audit procedure for new and locum staff, a lack of qualified staff to perform CRP POCT, and the competence of (locum) staff (better-safe-than-sorry approach, or unaware of possibility of CRP POCT). R proposed solutions such as better instructing the new and locum staff.
S03	In-person GFS. FF1 knew a couple of ECPs well in this group, as they were also involved as educators at the medical residency program. PG also participate in another national NH infection surveillance program. PG requested data to be split at the unit level, but according to the research team too few forms were registered to disclose these results safely. R asked for clarification on the top 10% benchmark.	FR2 not only explained the data but also tried to prompt PG for reactions based on earlier discussions in other GFS. FF1 complimented the group in the recap, noting that they had been doing very well: Furthermore, well, as we just discussed, overall it’s going very well, you could say. Especially compared to last year, you’ve managed to adhere to the guidelines even better. (S3) FF1 explained here to the group that the recap would be short due to the limited time available.	Very interactive session, with the CP initially being the first to respond. Rs did not expect so few recorded audit forms, which they found slightly disappointing. Rs believed that they had not recorded all cases. Discussions on different topics regularly ended with problems related to the audit procedure. The reactions to the data were predominantly positive. Although they scored 88% on the AB adherence rate, they were disappointed not to be in the top 10%. Rs found it interesting to see the reported reasons for intentional non-adherence and tried to contextualize/justify the non-adherent data. However, Rs suggested that most non-adherent cases had probably been “forgotten” to be recorded as “intentionally non-adherent”. The Rs shared difficulties that they encountered when ordering a CRP POCT.	During the discussion, the CP also prompted the group with questions (e.g., “Why do we prescribe more?”), which resulted in Rs sharing personal reflections/opinions, although this did not necessarily lead to change cues/ talk. FF proposed in the recap that the PG can discuss the appropriate use of CRP in another meeting and due to time constraints continued to the UTI part.
S04	In-person GFS with a PG where, according to FF1, “a nice distribution” of ECPs and NS were present. During the introduction, one of the ECPs introduced herself as the medical manager of the PG.	Fs answered questions from the R about ambiguities regarding the recording and guideline. There was no summary at the end of the LRTI section. Fs referred to the minutes that a R had been taking and asked a general question if all learning objectives had been included. It is unclear how the R could have answered that question: FF1: No, I think, yes, we’ve now reflected enough on that and formulated learning objectives, haven’t we? Those have also been noted. So, erm, are there any additional things you think you can take as learning objectives from this? Because then we’ll close the topic of respiratory infections, okay? FR2: Y, I think so, given the time as well, otherwise it will come in due time, yes. FF1: Yes, no, I think, yes, no further additions. (S4)	The CP and medical manager were often first to speak and emphasized that participation in the A&F program should be seen as a quality improvement tool. R questioned what the results meant when a large portion was “unable to assess.” Another R noted that if you leave out the cases due to lack of CRP POCT, the adherence rates were already near the Top 10%: R1: Well, if you take the CRP out of the equation, then we’re already getting close to the top ten [laughs]. (S4)	The CP was in the process of implementing the new CRP POCT device and informed with the Fs if there was any support for that. Furthermore, the medical manager mentioned feeling responsible for the insufficient introduction of the new staff to the A&F program and committed to paying more attention to this. R thought that it would also be valuable to discuss patient cases/chart reviews with each other more often, including outside of the A&F program.
S05	Hybrid GFS, which was initially unclear for the Fs, and it was only during the discussion about the first adherence rates that an on-site R remarked that they missed the presence of online R. An on-site cellphone was used to connect with the two online R, which made it difficult for the online R to participate. FF2 knew one ECP of the PG. CP of the group is a NP who used to collaborate with an ECP, but the appointed ECP left without a replacement. This was brought up after a question from FF2 regarding whether there was a driving force in the group. An ECP-R responsible for medication safety proposed to also become involved as CP. The ECP CP who had left requested additional analysis of the data for the presentation (differences between psychogeriatric and non-psychogeriatric care). When the Fs raised this, the group found it interesting but were unaware of any specific reasons for these additional analyses. R asked for clarification on how cases would be classified as “unable to assess.” R was unaware that the audit period had ended.	After FR2 presented the data, FF2 often responded first. FF2 contextualized the data for the group and explored reasons for non-adherence by making suggestions. PG ended up in a confusing discussion after FF2 asked if the local prescribing standard of LRTI had been reviewed against the regional resistance pattern. PG wondered if the pharmacist who they work with had assessed this and how reliable these assessments were. Many questions were raised about the added value for LRTI. FF2 concluded that the regional resistance patron might be more appropriate for UTIs than LRTIs and moved on to the next section due to time constraints.	Moderate interaction. R believed that non-adherent cases where AB were not initiated were “intentionally non-adherent” due to factors such as treatment restrictions but had been forgotten to be registered. R indicated that a CRP is often not done because it is “such a hassle” given that there is only one CRP POCT device at their main site. One R is involved in a new protocol to enable testing at each site. R3: Yes, previously we had no a CRP device at all, so that. (S5) R were not surprised about the high AB choice adherence rate, as similar results were also reported by their pharmacist. The PG mainly attributed this success to their local prescribing standard. A new occurrence this year was that 13% of the AB cases were prescribed ceftriaxone as adherent AB (n=2). R found this “actually not much” and no further remarks followed.	During the first discussion on treatment initiation adherence rates, R raised a change cue on CRP testing, stating that she never performs a CRP test. FF2 responded by stating that the CRP results would follow in a moment, although other R were responding on the issue raised at that moment. This original change cue was not further explored later on. Discussion around CRP POCT prompted a R to speculate how CRP POCT sampling works and whether it is possible to practice that on each other.
S06	In-person GFS in an NH where only the rehabilitation departments are participating. The group also sees opportunities to learn from the cases in the categories of “intentionally non-adherent” or “unable to assess.” CP would like further detailed information about the non-adherent audit forms so that they can review the charts for additional learning points. Prior to the meeting, the CP requested a stratification of the data at the unit level of their rehabilitation department, although the number of recorded cases was insufficient.	Fs emphasized the importance of learning. FF1 monitored this: when FR4 used words such as “right” and “wrong” FF1 reminded everyone that the goal is to learn and not to focus on whether something is right or wrong. Fs emphasized the low number of forms and the importance of effective recording during the audit period. Fs appeared to question whether these small numbers were realistic. FF1 used the benchmark results as input to reflect with the group. No summary of the LRTI part was made.	The CP was often the first to respond and it seemed like the CP talked more than the other Rs, especially at the beginning. Rs found the low numbers of AB prescriptions realistic (n=2), as proof of already prescribing AB very selectively in their PG. Rs found the audit recording procedure not to be optimal, and shared the problems that they encountered. Fs responded to the recording problems by explaining how the system should be used to avoid those problems.	When talking about reasons for non-adherence, a R raised a change cue on the appropriate use of the CRP test. FR2 stated that this would be discussed later in the CRP section. However, the specific issue was not rediscussed. Another R tried to bring it up again after the AB section. In the subsequent discussion, a comparison was made with the urine stick, and Fs identified a transition to continue with the UTI part. FF1: Yes, fine. I think that’s a nice transition, right? For the urinary tract infection. So, well. Let’s see what we have there, erm.. (S6) A change cue that was not addressed by Fs or Rs is the discrepancy between R mentioning that the local formulary follows the Verenso guideline but later on R mentioning that the Verenso guideline is possibly not very suitable for their rehabilitation department.
S07	In-person GFS, where NH’s rehabilitation departments participated in the AB topic and long-term care departments in the psychotropic topic to limit the registration burden. This selection was not clear to all Rs. At the LRTI recap, R wondered if they would be punished more. CP responded by stating that it is a waste of time and effort if forms are not filled out correctly and that it is therefore appropriate if it feels slightly like a punishment. R asked whether the results are based exclusively on registration forms or if other data sources were also used. They also questioned how one patient could have multiple recorded forms. Another R wondered about the duration of the audit period. An R found it “a bit strange” to change practice or treatment based on audit results that do not incorporate patient outcomes. CP saw himself as the driving force when FF3 asked if the group had one.	There was a lot of discussion about the recording of the audit forms. FF3 tried to limit the recording discussion. FF3 pointed out that other participating NHs would have also encountered these recording problems and comparing the PG’s own results to the average and top 10% benchmarks still looked less adherent. FF3 also explicitly stated that the discussion on the recording system had been covered now and should not be repeated again in the following parts. In the recap, FF3 started by asking: “What are your conclusions and do you say, ‘well we have to do something,’ or, ‘so be it?’” They then explored with the group ways to achieve the improvement goals that R shared. FF3 challenged Rs to make the goals SMART and reminded them that last year’s goal was already to acquire a CRP POCT device.	Moderate interaction. Rs believed that the number of recorded cases was low, feeling that they had not adequately recorded cases. Rs discussed the audit recording system extensively. During the CRP statement, R mentioned that insufficient testing is likely due to the absence of a CRP POCT device. The AB section began with R noting that they always adhered to the guidelines, but the locum staff, primarily trained in family medicine, did not follow the ‘correct’ guidelines. Upon reviewing the specific percentages of different AB, the group realized that their formulary was still not in accordance with the guideline. Some Rs believed that the formulary had been updated to align with the guidelines, while others thought that there were reasons for the discrepancy but could not recall them. CP concluded that they could “defend” their non-adherence rates in AB choice given that their formulary is different.	For changes to the formulary, they plan to ask a peer PG member (who was not present) at their regular PG meeting this afternoon. PG must be more aware of the effect of the departments with a high medical staff turnover (e.g. temporary medical residents or junior doctors) on the audit procedure, the specific guideline to use and the local prescribing standard. Regarding CRP POCT, Rs still wanted to invest in this but found it challenging to make it a more SMART goal, then planned to discuss it further at the next PG meeting. FF3 probably noticed that the PG felt they had to defend themselves concerning his questions about why it had not been purchased in the past year, reminding them: *FF3: Yes, this is not for me. It’s for you all. (S7)*
S08	In-person GFS that had been included in the off-site day of the PG. Only ECPs and their medical residents were present, together with their policy advisor. FF2 knew some of the group due to his teaching activities. The reason for this PG’s participation was to assess how they had improved over the past year. R asked clarification on the top 10% benchmark.	FF2 explored with the PG how to interpret the results and the possible causes for non-adherence. In the recap, FF2 concluded with a final remark that the PG is already addressing issues from multiple angles, whereby they should not tackle too much at once and rather focus on a few items, and see how the results are next year: FF2: Yes, that’s a also a good one. Yes, you’re already addressing many things from several angles. Yes. Yes, and you can’t tackle too many things at once; at some point, you don’t know, do you? Take on a few items and see what that does next year, I think that’s important. (S8)	Interactive session. Rs concluded that they were scoring average and were quite satisfied with this result: R2: But overall, we score quite average compared to the rest of… to the other organizations. (S8) They noted an improvement compared to last year and believed that next year, with the CRP POCT device available at more locations, the results would further improve. R mentioned that participating in this program made them more aware of the guideline and its implementation: R1: Yes, perhaps it’s simply the fact that this is the first time we worked with the A&F program, which could explain it. So, the fact that it was discussed in an pharmacotherapy discussion group and received more attention, so, yes, maybe you’re seeing that reflected here. (S8) R6CP: You can really see that learning from data, because these are data that we collected ourselves, are being discussed here. You already saw, in those two years we’ve been talking about it, that it led to better interventions, so it truly is a nice learning cycle, erm. (S08)	When discussing the reasons why cases were “unable to assess”, R already brought forward that only one site has a CRP POCT device, which makes it challenging to adhere to the guideline. R are working to invest in more CRP POCT devices. FR2 intended to contribute to the discussion by presenting the CRP audit results, but overlooked that the slides comparing treatment initiation rates with last year were shown before the CRP results, and the conversation switched to the comparison with last year. During the CRP segment, the group discussion focused more on which situations are appropriate for testing. In the recap, FF2 raised the question to the group about addressing potential non-adherence related to medical residents. Several solutions were discussed, leading to a proposal by R to more clearly specify in the onboarding documents the guideline and drug formulary to which the PG adheres. It was suggested that these changes could be integrated with the current revision of their onboarding process for new physicians.
S09	In-person GFS during an off-site day with the ECPs of the PG. An ECP of the PG who is also the network’s CP presented the data and led the discussion (co-F2). co-F2 held a preparatory meeting with FF1 and FR4. co-F2 prepared printed versions of the guideline’s flowchart for Rs to support the presentation and for Rs to use as a reminder at their desk. R saw the added value of the presented data, and for the CRP topic to measure again next year, for example. Rs wish to see the data in more detail, aggregated at the location level and office hours vs locum hours. co-F2 mentioned that these questions are valid and interesting but for now “global feedback” was presented, and that they would definitely encounter other data limitations. R asked for clarification of the top 10% benchmark. They were happy to see that they were in the top 10% concerning the AB adherence rates and wished to also be in the top 10% for the other results.	co-F2 presented the data systematically and at the same time gave an interpretation/explanation. co-F2 also shared what he found complicated about the data or “could not do anything with” due to the low numbers. Concerning the CRP data, co-F2 thought that it could not only be explained by the absence of a CRP POCT on locations, but the “actual usage is still an issue.” FF1 and FR4 occasionally assisted co-F2 with more detailed information on the data analyses or addressed issues raised during other GFS.	Interactive session in which Rs responded promptly to questions and other remarks. Rs appeared to follow/agree with the interpretation and explanation of the results presented by co-F2. Explanations for non-adherence were sought by R in the availability of CRP POCT (not available at every location), the locum services, and the audit recording system. After these issues had been addressed by the PG, R shared more individual actions: “Here’s hoping for the best, let’s do an antibiotic course.” Rs believed their threshold to fill in a form was perhaps too low and all those forms that could not be assessed could have better been not recorded. Furthermore, audit recording problems such as completing audit forms and forgetting to record cases as “intentionally non-adherent” were brought forward. In response to the introductory statement about the CRP POCT, R already reflected and provided different practice examples.	co-F2 announced during the introduction that he planned to review the findings more critically and in depth with a colleague to ensure a follow-up of today’s session. The R within the PG responsible for the implementation of the CRP POCT devices planned an action to inquire within the organization on the progress. FR4 wondered how the PG will monitor progress on the CRP POCT. R responded that it would be addressed in their next PG meeting. R suggested that the AB selections in the formulary for LRTI should also be based on regional resistance patterns. The discussion that followed led to another R responsible for infection prevention within the PG explaining that this matter had already been addressed in a regional infection prevention meeting and that it is not meaningful for LRTI to do.
S10	In-person GFS. PG was especially interested in the CRP results because they had acquired a CRP POCT device in the summer before the new audit period started. PG also set a goal for this topic. Some of the R reviewed the presentation that was sent in advance, while others did not have time for that. Rs became more aware of the possibility to check the “intentionally non-adherent” box on the audit recording form. The first part about CRP testing was not audio recorded on tape.	Fs both emphasized the low number of recorded cases multiple times during the presentation. The issue raised around the presenting symptoms included in the guideline seemed to be approached by Fs with a focus on the audit recording system. FF3 also seemed focused on the question of whether Amoxicillin is in their emergency supply inventory, as well as the onboarding procedure of new medical staff.	R recognized themselves in the presented data and did not seem bothered by the low number of cases. The first response by R as a reason for non-adherence was related to CRP testing, to which FF3 informed the group that those issues would be discussed later. The adherence rates on treatment initiation were slightly disappointing for the R: “I am somewhat disappointed” (S10). They especially found the presenting symptoms on the flowchart difficult, as these did not always match with practice. This issue had already been brought up in the introductory statement discussion on the flowcharts and recurred in almost every discussion in the GFS. R also tried to understand why these cases were captured in the audit recording system. Fs took the time to explain this. CRP testing was not fully captured on the audio recording, but in the last part of the discussion Rs talked about CRP results and their clinical value.	FF3 pointed out the number of Amoxicillin prescriptions in the data, and then asked about the emergency supply and suggested a potential improvement to remove Amoxicillin from the emergency inventory. An R initially responded they would look into this. Later, when FF3 reiterated this action, another R shared that Amoxicillin is not part of their emergency supply. The suggestion by FF3 to pay attention to their onboarding procedure led to an idea from R to include more information on the guidelines that new medical staff should use. Furthermore, Rs saw possibilities to adapt the already planned clinical training to the results of this GFS, and decided to organize this training not only for the junior doctors but also for the supervising physicians. They also wanted to print the flowcharts as pocket-size cards as a reminder for them on the work floor.
S11	Online GFS with an NH organization that participated in the A&F program with only one location. Only the medical team from that location took part. Two junior doctors and a non-medically trained manager from the PG were present, while the ECP and nurse practitioner were called away for an emergency situation. FF1 appeared to be very conscious of the fact that R were junior doctors, as she mentioned this several times. During the first introductory statement, an R's camera remained off. However, it was switched on after FF1 remarked that it would be better if everyone could see each other. Following a remark by R about the delivery problems concerning Augmentin in their NH region, it became clear that R thought the audit period was still ongoing.	FR3 suggested at the start to move through the LRTI part quickly to allow more time for the UTI part, due to the low number of registered LRTI cases (n=8). During the CRP section, FF1 focused on the absence of a CRP POCT device in the NH. FF1 emphasized the importance of including in the minutes that having a CRP POCT device is essential for adhering to the guideline. AB adherence rate was 100% based on three forms. FF1 took a moment to explore the local formulary that they used, and Rs shared that the local formulary had been recently updated, but they were uncertain whether it had been done based on the Verenso guideline. In the recap of LRTI, FF1 summarized the findings and then asked Rs if anything was missing. FF1 was positive and encouraged R to continue in this manner. FF1 did not actively involve the manager in previous parts due to the manager’s non-medical background. FF1 explained this to her before inviting her to contribute to the summary if needed. FF1 proposed to Rs to report today’s findings/minutes at their next regular PG meeting.	Interactive session. R thought that the low number of forms was due to the infrequent occurrence of LRTIs. R mentioned that they had sometimes cancelled an audit recording form because the suspicion was an upper respiratory tract infection. Based on the responses of Rs to the introductory statement about CRP, it appeared that they were unaware that the guideline recommendation for severely ill patients suspected of LRTI is to start antibiotics immediately rather than performing CRP testing. This possible knowledge gap was not explored.	There are no change plans.
S12	In-person GFS. One ECP-R knew FF3 because he did his medical residency training in an NH in which FF3 was the residency program director. PG participated in both AB and psychotropics topics. R mentioned that by participating in this A&F program, they increased their awareness of adhering to the guideline.	When the Rs were discussing the audit recording system, Fs tried to redirect the discussion by asking, for example, which CRP adherence rate they would be satisfied with next year. However, R answered with doubts about whether the audit procedure can capture these improvements. Due to time constraints, Fs did not explore all issues raised by Rs. The uncertainty in the group regarding the Amoxicillin prescription and their trust in the data prompted FR2 to check the data after the GFS. Upon confirming that the medical GP resident prescribed it, it made more sense to the group. FF3 asked Rs to summarize what is going well and what needs attention. FF3 explored with the group how they would approach the points that needed attention.	Rs were interested in the category of “intentionally non-adherent” and “unable to assess” to help them to better understand the data. From the data, R concluded that the low adherence can be explained by the lack of a CRP POCT device during the first part of the audit period. CRP POCT had only recently been introduced at their main site. When discussing the CRP adherence rates, Rs concluded it is clearly still an improvement goal. Rs also took the time to share their reflections on their experiences with the implementation process of CRP POCT and the improvements that they have already made. Rs also discussed issues that they encountered with the audit recording system. Fs addressed these issues in detail. R questioned whether the 6% difference in adherence rates shown in the comparison with last year was not just a coincidence. In the AB section, Rs expressed surprise that someone prescribed Amoxicillin. Rs could not believe who did this, and they were inclined to believe this was a recording error rather than reality, although R suggested that the medical GP resident was perhaps the prescriber.	In the recap section, the group formulated the following action plans: -The R responsible for the medical GP residents will pay more attention during the onboarding to the difference between the GP and NH guidelines. -They will print out the guideline’s flowcharts and hang them at their desk. -They will monitor the CRP testing for six months during out-of-office hours, and after the evaluation address any issues with the locum agency that cover their on-call shifts. -The Rs on the remote site will monitor the frequency of indications for CRP POCT for six months to see if a device is also needed at their site. Rs indicated that in the week following this GFS, the PG’s quality visitation is scheduled by the professional association of ECP. Some of these plans can be incorporated into the upcoming year plan.
S13	In-person GFS. They specifically requested that their antibiotics data could be stratified between moderately and severely sick patients with LRTI to align with their regional formulary.	FR1 skipped the comparison slide showing last year’s adherence rates, because only two cases were registered last year. FR1 also skipped the introductory statement on CRP usage as it had already been discussed in the previous introductory statement. In the questions and remarks placed by FF2, FF2 used examples mentioned in previous GFSs. In the recap, FF2 summarized the improvement points for the group, placing more attention on recording cases as “intentionally non-adherent” and re-evaluating the decisions made regarding CRP testing in their practice.	Interactive session. Rs were surprised that the “intentionally non-adherent” rates were not higher. Given that the PG has a regional formulary/prescribing standard that differs from the guideline on CRP testing and recommended antibiotics, Rs repeatedly concluded during the presentation that they should remember to record their cases as “intentionally non-adherent.” Several Rs also discussed in various ways whether a CRP POCT (device) would be useful in their practice. R found it difficult to decide this based on the presented data. Rs also mentioned the challenges of implementing CRP POCT across the numerous NH locations within the organization.	After the recap, Rs began to question the reasons for the regional differences from the guideline, and whether the decision for no CRP POCT should be re-evaluated. No R could share the exact reasons for the regional differences, but an R mentioned there is a regional meeting planned at the end of the year with the experts of the regional standard. This R, who is responsible for the meeting, asked everyone with questions to email them to her so she could collect them for the meeting. In the meantime, for the next audit round, PG decided that every recorded LRTI case should be recorded as “intentionally non-adherent” given their deviating formulary/standard.
S14	In-person GFS with a PG that participated for the first time. They saw this as a baseline measurement but were not exactly certain if all locations took part in the audit, especially the remote location that has no CRP POCT device. R asked for clarification on the top 10% benchmark.	Fs directed the group towards specific topics, and among other things the recording problems were discussed, but in other moments they let the group freely discuss. Fs asked the group what their take-home message is from the LRTI presentation.	Interactive session, in which the CP had a lot of input. Rs shared audit recording problems when the results were shown on “unable to assess.” R interpreted the “intentionally non-adherent” cases as adherent, which brought their adherence rates closer to the top 10% benchmark. R also considered the possibility that some cases were simply forgotten to be recorded as “intentionally non-adherent.” In the AB section, the PG discussed all of the prescribed drugs and reasons for starting non-recommended ABs. Critical remarks were made by R regarding the relatively high proportion of Ceftriaxion prescriptions, although this issue was not further explored.	During the LRTI recap, Rs expressed the desire to improve the audit recording process for intentional non-adherence, focusing particularly on reasons for initiating antibiotics.
S15	In-person GFS. FF2 knew a couple of ECP-R from back when they were still medical residents and FF2 was their teacher. Following the reactions and questions of R, it seemed that they (also) viewed participation as a form of assessment. R asked for clarification on the top 10% benchmark. R initially assumed that locations without a CRP POCT device would not be included in the CRP adherence rates.	FR3 likely felt time pressure as both AB and psychotropic topics were presented in the GFS. Especially early in the GFS, Fs answered many questions themselves. Later, Fs gave more room for the group to engage with each other. Topics postponed by Fs for later were not actively revisited by the Fs. There was no summary during the recap, but Fs mentioned additional actions that the PG could undertake to improve the audit recording.	Interactive session in which several clinical practice issues were addressed by the PG, but most discussions also included mistakes and problems related to the audit recording. Rs spoke openly to each other, as well as with room for humor, and reminded each other of previous agreements made within the group about the audit recording procedure and CRP testing.	During the discussion on reasons for non-adherence, the issue of CRP testing at their smaller locations was raised. Fs responded by stating that CRP would be addressed later on, but this specific issue was not readdressed. Rs perceived potential audit recording problems as explanations for non-adherence. Fs proposed solutions themselves to the problems raised and then moved on to the next section, possibly due to time constraints.

*A&F* Audit & Feedback, *CRP (POCT)* C-reactive protein (point-of-care testing), *ECP* elderly care physician, *GFS* group feedback session, *GP* General practice, *NH* nursing home, *PG* physician group, *GFS* group feedback session, *CP* contact person of the physician group, *AB* antibiotics, *LRTI* lower respiratory tract infection, *UTI* urinary tract infection, *co-F* co-facilitator, *FF* faculty facilitator, *FR* research facilitator, *Fs* facilitators, R(s) recipient(s)

## Prerequisite activities

### Organization

Among the fifteen group feedback sessions, twelve were in person, one was hybrid, and two were held online. In-person group feedback sessions seemed to lead to the best group dynamics. Although the numbers of hybrid and online sessions were limited, there was a difference in the interaction and engagement between the hybrid and online sessions and the in-person sessions. Digital support appeared to be an important factor in this process, which was also indicated as an area for improvement by facilitators on the evaluation questionnaire. When some participants were gathered together behind a single screen, it was difficult for the facilitator and other participants behind their single screen to hear and understand what was happening among these groups of participants.

The group size also seemed to play a role in interaction and engagement, for both online and on-site sessions. Compared with the online session with twenty participants (S02), the online session with three participants had better interaction and engagement (S11). The on-site group feedback sessions with fewer than eight participants were the most interactive (S04, S06, S10-S14). Facilitators’ evaluation also revealed that in larger groups it was not possible to engage all participants in the group conversation (S01-S03, S07). Interactive group feedback sessions with more than eight participants were seen in physician groups where the facilitators were already acquainted with several participants (S03, S08, S09, S15) and/or the physician groups had a prominent contact person who actively participated during the group feedback session (S03, S04, S07, S09). The contact person of S03 prompted the group directly with several questions, which led other physician group members to share more reflections.

### Relationship building

The relationship between the facilitator and the physician group members played an important role in the dynamics of the group feedback sessions. The majority of the participants were not acquainted with the facilitators. At the start of the session, the facilitators held a plenary introductory round with all participants, except in the two sessions with larger groups, where the facilitators only introduced themselves to the participants (S01, S02).

The relationships among the participants and whether they felt they were in a safe environment seemed to influence the dynamics of the session. When the atmosphere seemed relaxed with participants making jokes, showing interest, and being willing to speak up, there tended to be more mutual interaction between the participants (S03, S14). According to the facilitators’ evaluation, S01 also had an ‘open’ discussion and in S06 the participants showed an ‘open’ attitude, strong enthusiasm, and a positive atmosphere. A facilitator commented that the interaction was difficult in the physician group S02’s feedback session, possibly due to the meeting being online and probably also given the apparent absence of a connection among the participants within the group.

To strengthen the educational focus of the physician groups, the A&F network coordinator encouraged groups to establish learning objectives and/or additional data analysis questions during the audit period, to which nine contact persons responded. Some of the groups requested analyses that were already part of the standard A&F: one group that participated for the first time requested a baseline measurement, whereas others requested a comparison of their adherence rates with those of the previous cycle or other participating groups. A specific learning objective was regarding LRTI diagnostics and specifically the effects of the CRP point-of-care testing (CRP POCT) device implementation in an organization (S10). Some of the additional data analyses could not be performed with the available audit data. For example, physician groups S03, S06, and S09 asked for adherence rates at a more detailed level than the whole physician group (e.g., location level), although the number of registered cases was too small to share the results.

In two physician groups, the contact person was willing to facilitate their own group feedback session. S01’s contact person fulfilled the role of a faculty facilitator during the session, which was conducted together with a research facilitator. S01’s contact person was an elderly care physician who led group feedback sessions in the first cycle as a research physician. S09’s contact person was an elderly care physician who is the chairperson of the physician group. S09’s contact person presented the data and led the discussion. The research and faculty facilitators were present in a supportive role in the group feedback session. Both co-facilitating physicians had a pre-meeting with the facilitators to discuss the audit results and the presentation and they made additional preparations for their own group feedback sessions. S01’s co-facilitator conducted additional data analysis on their prescription data. S09’s co-facilitator printed out the guideline flowchart and planned a follow-up meeting with a colleague in advance.

### Question choice

Question choice concerns whether the topic is right for A&F and important to participants, and whether the data sources and limitations are clear. The reactions across group feedback sessions revealed that physician groups recognized the added value of this A&F program and its topics (S08, S12).

*R6CP: You can really see that learning from data, because these are data that we collected ourselves, are being discussed here. You already saw, in those two years we’ve been talking about it, that it led to better interventions, so it truly is a nice learning cycle, erm. (S08)*

The usefulness of the feedback information concerning the use of the CRP POCT device was questioned most often. Some nursing home organizations had no CRP POCT devices, whereas others had locations with and without CRP POCT devices. This raised questions among participants about whether locations without CRP POCT devices should be excluded from the data (S01, S09, S15). Some participants questioned the data by commenting that the audit measures did not reveal whether non-adherent medical care affected patient outcomes, either generally or specifically in relation to CRP POCT (S07, S12, S13).

Based on the participants’ questions and remarks, it became clear that the knowledge about the data sources used and the limitations of the audit results varied within each physician group (S01, S02, S05-S07, S09, S13, S15). Although the contact person of the physician group had made a decision prior to the session concerning the antibiotic topic and the nursing home locations included in the audit, this was not always clear to the physician group (S01, S07, S14). This lack of clarity was partly due to some participants joining during the audit period or not being involved in the audit at all (S01, S02). Even among those involved in the audit process, there was a lack of understanding of how their audit recordings translated to the audit results (S01, S03, S04, S06, S07, S09, S13, S15).

Requests for additional in-depth analyses were also made during the group feedback sessions (S01, S06, S09), including requests to receive data at the patient level so that the group could review the records for further learning (S01, S06). One of the physician groups suggested planning chart review meetings outside of the A&F program (S04).

### Data representation

Based on the participants’ reactions, the majority of the graphs – along with the research facilitator’s explanation – were easy to understand. The top 10% benchmark was a difficult part of the graph for participants to understand and often needed additional explanation (S03, S08, S09, S14, S15).

### Audit and group feedback session

#### Facilitation

Although sharing a common professional background (Supplementary Table 1, Additional File 1), each facilitator brought a unique perspective and individual expertise to the group feedback sessions. Comparing the group feedback sessions revealed both similarities and differences among the facilitators, as well as among sessions led by the same facilitators. All of the facilitators were aware that numerous topics had to be covered within a limited amount of time. Occasionally, the facilitator ended discussions and moved to the next part due to time constraints (S01-S05, S12). In other group feedback sessions, the facilitator informed participants from the start not all topics could be addressed in detail due to limited time (S02, S15).

Unexpected events such as a power cut (S01) or an on-site meeting turning hybrid after the start (S05) required facilitators to improvise and be flexible during the group feedback session. Furthermore, reactions of participants prompted the group session to topics that were scheduled for later in the session. Facilitators handled these situations differently: sometimes this was addressed directly (S03, S04), while in other instances facilitators attempted to reserve the discussion for the intended part of the presentation (S05, S06, S10, S15). These unrecognized change cues were often not readdressed or appeared to be discussed less elaborately, especially in situations where the facilitator did not recognize these reactions as change cues (S06, S15).

All of the facilitators were able to lead the conversation in certain directions by – for example – highlighting certain results or asking questions to the group to move the discussion towards a change cue or help the physician group to develop action plans. In several group feedback sessions, the facilitator made the recording for the audit a subject of discussion (S01, S02, S14), even repeatedly in different parts of the session (S06, S10). In other group feedback sessions, the facilitators tried to steer the discussion away from the audit recording system (S07, S11).

*FF3: Yes yes, well. Erm, yes, I think we should move on, and this point [audit recording system] comes up in all three topics. We’ve kind of covered it now. So, we don’t need to address it with the other two topics [urinary tract infections and psychotropic medications]... (S07)*

*FF1: Alright, but let’s move on, because this is more about recording than about, erm, yes, content. (S11)*

A comparison of the co-facilitated sessions S01 and S09 with the other sessions revealed that the co-facilitators leveraged their experience in the physician group and the context/setting in which the group practiced. The co-facilitators were more direct in contextualizing the results by – for example – giving examples and dismissing irrelevant results or remarks from the participants. The ‘regular’ facilitators did not benefit from being part of the physician group, although they connected with the group by drawing on their own experiences or those shared by participants in previous sessions (S02-S05, S07-S10, S12, S13, S15).

### Reactions to data

Overall, groups reacted positively to the audit data, especially if they were among the best performing. Physician groups experienced achieving the top 10% as a ‘pat on the back’ (S09). Additionally, participants in several physician groups expressed an aspiration to be among the top 10% performing nursing homes (S03, S09, S10). When the data were not favorable, they were often met with doubt by participants who did not recognize themselves in the data. Within groups with relatively low adherence rates, participants asked whether they “*would be punished more”* (S07, S15) and felt that they needed to defend themselves (S07). Low numbers of registered cases were often met with disappointment. S06 saw these low numbers as confirmation of their selective antibiotic prescribing policy.

*co-F2: [...] Is the AB choice according to the guidelines? Erm, well, yes, you’re among the top ten.*

*R8: Yeah.*

*R5: Yay!*

*R4: There’s a first time for everything. [laughter among the group]*

*R6: We’ve got that one to a tee! [laughter and talking over one another] (S09)*

### Understanding and questioning

The participants asked questions to better understand the data in relation to the audit procedure and questioned some of the non-adherence categories:

*R2: I have a question, if that’s okay, erm, because I think it would look very different if you didn’t include those 36 [items that were unable to assess] in the analysis. (S02)*

Questions about the audit procedure addressed issues such as the required level of suspicion on LRTI for inclusion in the audit, audit recording forms that were not immediately completed by the initiator, and the importance of recording cases on the form as being intentionally non-adherent to the guideline recommendations. (S01, S02, S04-S07, S09, S12-S15).

### Justifying and contextualizing

To justify or contextualize the data, physician groups mostly look at external reasons beyond their direct control as physicians. Examples of such external reasons are physicians in training and (external) locum physicians in the evening, night, and weekend shifts who are not always familiar with the nursing homes’ guidelines (S01, S02, S07, S09, S12), changes in the physician group (S02), a specific patient population within the nursing home (S04, S12), or consultations with medical specialists who recommend different treatments (S01, S04).

### Reflecting, sharing practices, and discussing evidence

The amount of reflections, sharing one’s own practice problems or discussing the evidence for best practices varied across the group feedback sessions. The reflections and challenges shared by participants regarding diagnostic uncertainty and the use of CRP testing were recognized by the other participants. The responses were mostly supportive of each other and occasionally critical (S06, S09, S10, S15). Notably, these critical remarks sometimes occurred the second time an issue was readdressed (S09, S10). In several instances, reflections and discussions on clinical topics in the group conversations circled back to problems related to the audit recording procedure or other external causes.

### Change cues, change talk, and action planning

Change cues and change talk occurred in physician groups most often about topics with low adherence rates (See Supplementary Fig. 1, Additional File 1). Therefore, given the relatively high adherence rate across the physician groups for antibiotic choice, fewer change cues and less change talk were observed on this topic. Change cues and change talk on the treatment initiation measure focused more on possible mistakes regarding the audit procedure than those in clinical practice. Change cues and change talk concerning CRP testing ranged from clinical indications and evidence around CRP testing to practical and logistical barriers. The introductory statements were sometimes sufficient reason for participants to start an in-depth discussion before the data was shown. Moreover, after the data had been shown on – for example –*reasons for non-adherence*, this already led participants to raise change cues on CRP testing (S01, S04-S06, S08, S09, S12, S14, S15).

Action plans formulated during group feedback sessions were often related to the audit recording procedure, knowledge deficits of (new/locum) staff, and the purchase of a new CRP POCT device (Table 3). In most groups, the feedback session alone did not provide sufficient time to develop action plans for changing treatment or prescribing practices. In these situations, groups ended up with an action to discuss their plans further in a subsequent meeting (S02, S07). Facilitators also parked change cues/talk with the suggestion to further explore them in another meeting (S03, S07-S09, S11, S13).

**Table 3:** Example of action plans

• Acquiring CRP POCT device (S01, S04, S09, S13)
• Providing instructions to the physician group on how to use the CRP POCT device (S01, S02, S05)
• Physicians on the remote site will monitor the frequency of indications for CRP POCT for six months to see if a device is also needed at their site. (S12)
• Improving the onboarding procedure for new medical staff (S04, S07)
• Instructing the general practitioners in training and locum staff to use the appropriate guideline in nursing homes (S08, S12)
• Discussing patient cases to improve quality of care (S04)
• Improving knowledge about the audit procedure (S02, S05, S07, S09, S13-S15)
• Revising the local formulary (S01, S07, S13)

Action plans proposed by facilitators were rarely adopted immediately but could provide groups with new ideas for other actions or were rejected by the group following a short remark or extensive group discussion (S10). Facilitators especially proposed action plans on how to handle audit recording problems, which were met with mixed feelings (S06, S07, S10, S13-S15). The participants felt that the facilitator’s suggestions were not always suitable for their group.

### Comparison of all physician group feedback sessions

A comparison of the different action plans of the fifteen physician groups revealed that specific actions were formulated in physician groups S01, S04, S05, S07-S10, S12, and S13. These actions included acquiring a second CRP POCT device, updating the formulary, improving the knowledge of new physician group members, monitoring the implementation of a CRP POCT device, organizing training on how to use the CRP POCT device, and gathering additional information before changing clinical practice. Factors that appeared to contribute to these specific actions included the responsibility taken by a physician group member or their motivation to address a specific issue, which might have been influenced by the key role that the member held within the physician group (e.g., responsible for antibiotic stewardship, the local formulary, or the implementation of CRP POCT devices), as well as the ability to integrate action plans with existing initiatives.

Physician groups S02, S03, S06, S11, S14, and S15 identified a range of possible causes of their current performance without reaching any action planning. Factors that appeared to contribute to this included physician groups’ adherence rate to the guidelines, the non-attendance of relevant physician group members, time pressure, a prominent facilitator, and strong focus on the audit procedure. In groups S03, S06, and S14, (parts of) the audit results might not have provided a direct reason for the physician group changing their clinical practice, as they perceived their results positively. Although the participants in these groups still shared practice dilemmas with each other, the conversation on these issues did not evolve into change talk and action planning. Group S11 planned to readdress the audit results in their regular physician group meeting, since the session was only attended by the two junior doctors and their non-medical manager. In groups S02 and S15, time pressure experienced by the facilitator from the start might have been a reason why plans were not discussed in more detail. The faculty facilitator in both sessions was prominent in suggesting actions instead of letting the group formulate possible actions. Participants in both groups also had a strong focus on the audit procedure.

In some of the group feedback sessions – both with and without action planning during the session – another meeting was suggested by the facilitator or the group to discuss further action planning (S03, S07-S09).

The facilitators’ evaluation questionnaires indicated that formulating specific action plans was challenging in several group sessions (S01, S02, S05-S07, S09, S12, S14, S15). Facilitators had different approaches in all parts of the group feedback session. The facilitators also used various approaches in assisting groups to develop action plans, which were especially evident during the recap section. At times, facilitators used open questions to explore what changes were needed, followed by a coaching-oriented approach to guide the group on how to achieve their goals, offering suggestions and thinking along with them (S05, S08, S10, S14). In other sessions, the facilitators asked follow-up questions to make the action plans as specific as possible (S07, S11, S12). In some sessions, the facilitator summarized the main findings and proposed solutions to the group, which generally resulted in approving responses (S02-S04, S13, S15). The approach used appeared to be influenced by the group dynamics, the responsiveness of the group, and the extent to which physician group members took initiative in addressing an action.

## Discussion

### Principal findings

The aim of this study was to gain a better understanding of how physician group feedback sessions in nursing homes were conducted and resulted in action planning, using the Calgary A&F Framework and Cooke’s conceptual model of physician behaviors [19, 20]. Enhancing the group’s ability to explore opportunities for improvement can be achieved by focusing on building trust in the data and the mutual relationships among group members and facilitators. The pattern of reactions and behaviors when physician group members receive audit results together with their peers is in line with Cooke’s conceptual model of physician group behavior [19]. Whether or not a group moves to action planning depends on many factors of the various A&F designs and implementation components that also interact with each other. We have added ‘organization’ – such the type of meeting (online, hybrid or on-site) – as an important prerequisite component. The lessons learned to improve the group feedback sessions in our A&F program are summarized in Table 4, and the key lessons are discussed here.

**Table 4:** Lessons learned from physician group feedback sessions in nursing homes

• Train facilitators to engage participants and to effectively lead group discussions to action planning
• Consider who among the physician group should participate in the group feedback session
• Report audit results at both the physician group level and a more detailed level of feedback
• Prefer in-person group feedback sessions. If physician groups plan an online/hybrid meeting, another setup is needed with adequate digital support
• Focus on important areas with room for improvement and prioritize topics prior to the session, both in accordance with the physician group
• Show the achievable benchmark in an absolute manner, such as the top three physician groups. This may be easier to understand than the top 10% physician groups
• Encourage co-facilitation by a physician group member to lead the group discussion
• Suggest specific actions to the physician group to formulate as an action plan to improve their practice

### Comparison with prior work

Our findings suggest that the combination of recording additional audit data, receiving the information in a presentation, and discussing it with peers makes physicians more aware of the guidelines and their implementation in practice. Among the various behavior change techniques, feedback on behavior, social comparison, and instruction on how to perform the desired behavior are among the most common [28], whereas merely presenting an A&F report does not effectively reinforce behavior change [29]. Moreover, combining multiple behavior change techniques aligns with recommendations on delivering A&F to promote practice change [8].

In addition to Cooke’s framework [20], we identified another important component in group A&F sessions. Organization seems to play an important role in prerequisite activities, with the type of meeting (online, hybrid, or on-site) influencing the success of the group feedback session, for example. The use of in-person presentations without appropriate adaptations for online or hybrid settings could potentially contribute to reduced levels of interactivity and engagement. Online and hybrid physician group feedback sessions require a different setup than in-person group feedback sessions. In online meetings, it takes longer to establish relationships, the social presence of participants is weaker, and interactivity is more difficult due to having fewer non-verbal cues and a dependence on audiovisual technology. It is therefore important to focus more on relationship building in online meetings. Blanchard and McBride (2020) [30] reported that a lack of social presence limits who is available for interaction. If everybody is behind their own screen with their camera on, everybody can be evenly seen, which increases social presence and therefore the chances of interaction. In-person meetings seem to be most effective to establish relationships and increase interactivity. According to Muller-Schoof et al. (2022) relationships that include a safe team climate and a good team spirit are important facilitators in learning in nursing homes [7]. Future online and hybrid sessions require modifications and appropriate digital support.

Our findings confirm the importance of question choice in the effectiveness of A&F group feedback sessions. The importance and relevance of the audit measures are key variables that influence physicians’ acceptance of the feedback and their intention to change their behavior [31]. Given that physicians might feel a discordance between patient-centered ideals and quality improvement interventions [4], learning objectives should reflect the physician’s priorities. Previous research has shown that group A&F might be more amenable by focusing on a single topic area, ideally identified by the group [20, 32, 33]. Also, prioritizing themes within the group feedback session and focusing on certain action plans can contribute to quality improvement [34].

According to Crawshaw (2023) [28], feedback that recommends specific (e.g., if–then plans) rather than general actions is more likely to be effective. This is also in line with other research in which an action implementation toolbox with suggested actions and materials helps to translate intentions into action [35]. Furthermore, sharing examples of effective actions taken in response to A&F can inspire participants [36]. In our research, facilitators prompted the group with possible actions (more on audit procedures than LRTI topics), which led to groups either discussing them to adapt to their local circumstances or rejecting proposals. One common action proposed by physician groups was to schedule a follow-up meeting to discuss further action planning, especially when changes to clinical practice were involved. Similarly, multiple feedback sessions were often necessary in other A&F studies [32, 37, 38]. A follow-up meeting could also be useful to evaluate the action plans carried out, discuss what successes have already been achieved and what follow-up steps are needed [34]. To some extent, physician groups participating in consecutive years on the same topic have a follow-up meeting with the new A&F round.

### Key lessons learned

#### Train facilitators to engage participants and to effectively lead group discussions to action planning

Facilitators play a crucial role in supporting groups interpreting audit results, guide discussions toward sharing change cues, and explore potential actions. Similar to other studies on group feedback sessions, the (co-)facilitator’s characteristics significantly impact data interpretation, group discussions, and action planning [20, 32, 39]. Change cues are more likely to lead to change talk when the facilitator acknowledges and promotes dialogue around them. The Calgary A&F Framework recommends that having a physician group member as a co-facilitator enhances this process [20]. However, in our study, this dynamic was not evident in the co-facilitated sessions compared to those sessions led by our ‘external’ faculty facilitator. Nonetheless, since in some physician groups our faculty facilitators had prior relationships with some group members, they were not entirely unknown to each other. Desveaux et al. (2023) [32] argued that group feedback sessions might be more effective when led by an external facilitator. Regardless of whether an external facilitator or co-facilitator is used, facilitators must have knowledge and understanding of the nursing home context, which all of our faculty facilitators and co-facilitators possessed. Additional training – as proposed by both Cooke (2018) [20] and Desveaux (2023) [32] – could further enhance the effectiveness of group feedback facilitators. Facilitators’ training often involved Cooke’s (2018) findings [20] together with Sargeant’s (2015) R2C2 model [10], which focuses on building relationships, exploring reactions and understanding, and engaging participants in change talk to develop achievable action plans. Facilitators’ training might also benefit from incorporating motivational interviewing techniques, which helps to elicit change talk in ambivalent participants and support self-efficacy in action planning [40–42]. Such training can provide facilitators with the necessary skills to effectively lead group feedback sessions.

#### Consider who among the physician group should participate in the group feedback session

Our results demonstrate – like in other research – that the effectiveness of action planning is influenced by the number and type of participants [43, 44]. Smaller groups tend to foster more interaction, with members being more strongly influenced by those with whom they engage [43]. In contrast, larger groups are often swayed by the most dominant speaker. However, this dynamic is not necessarily a drawback, especially as key physician group members can effectively leverage their social influence and possess the capabilities of an educator or change driver, thereby significantly facilitating change [29, 45]. Following this, we found that in group feedback sessions where a key physician group member was absent, follow-up action was to reach out to this member. Given that group feedback sessions that included key members led to more concrete action planning, it might be useful to ensure that key physician group members attend the group feedback session. We also found that having an contact person or manager who is actively involved or present can positively affect interactions and action planning. Future research could explore this further.

#### Report audit results at both the physician group level and a more detailed level of feedback

The participants in our study received feedback at the physician group level. However, several physician groups requested feedback on a more detailed level – at either the location, unit, physician, or patient level – prior to or during the group feedback sessions. Previous studies on group feedback sessions have provided individualized feedback reports [20, 32, 38]. Desveaux et al. (2023)’s [32] group feedback session was intended to focus on identifying organizational level actions that the team could take to improve the quality of care, while team members received individualized practice reports. They detected a tension between the perceived organizational focus of the group sessions and the individual-level nature of the data. Since aspects of team functioning are associated with improved patient outcomes, looking at both the individual and group level might be beneficial [46]. Other research suggests that individual – as opposed to team-wide – feedback, increases recipients’ acceptance and intention to develop action plans [31, 47, 48]. Physicians often struggle to understand how aggregate practice-level data reflect their individual work [49]. Moreover, earlier studies indicate that self-assessment among professionals – including physicians – is often unreliable, with the poorest performers being the least accurate in assessing their own performance, reflecting a phenomenon known as the Dunning–Kruger effect [50–54]. In our study, the audit numbers per physician were currently too small to disclose individual results. However, depending on how care within a nursing home is organized, aggregating the data at a more detailed level might be both feasible and insightful.

#### Strengths, limitations, and future research

To our knowledge, this is the first study to evaluate facilitated physician group feedback sessions in nursing homes. A specific strength of our A&F program is that physician groups can record intentional guideline non-adherence and the reason not to follow the guidelines. Another strength of the study is the data triangulation, using qualitative data from questionnaires filled in by both facilitators after each session. These data could be used to support our findings or detect deviant cases. A final strength is that rather than adopting a general framework or inductively developing a new one in our data analysis, we used a specific existing framework and model to evaluate the group feedback sessions. This approach strengthens our study by building on the Calgary A&F Framework and Cooke’s model of physician behavior, both of which were especially developed for physician group feedback sessions.

One limitation of a deductive approach is the risk of not capturing new concepts. To mitigate this, we maintained an open focus during the analytic process, which led to a new theme within the framework and additional sub-codes in existing themes. Some of the sessions were coded individually by two researchers, while the rest of the transcripts were checked by a second researcher. Any discrepancies were discussed. Another limitation of the current study design is that we did not have independent in-depth observations of nonverbal interactions and group dynamics. The free-text responses collected on the evaluation questionnaire by facilitators leave the impression that nonverbal signals influenced group dynamics, group discussions, and, most likely, resulting action plans. Future studies would benefit from an observer present during the group feedback sessions [34]. Final limitation of this study is that we did not follow up with the action plans of the physician groups and rather evaluated the group feedback sessions based on their intentions for change. Nonetheless, studies suggest that the intention for change can predict actual subsequent practice change [55–57].

Future research could explore how inter-facilitator differences in relation to context – such as group composition, size, and type of session (online, hybrid, on-site) – play a role in the effectiveness of an A&F session. Additionally, research could focus on the effects of audit and group feedback sessions on formulated action plans and their implementation, as well as whether this is more effective than other types of A&F. Finally, future research could focus on whether recommended action plans by – for example – the facilitator are more often implemented than those developed by the physician group.

## Conclusions

This study shows that across the group feedback sessions in our A&F program for physician groups in nursing homes, the same variety in the development of action plans was observed as in other audit and group feedback session studies in other settings. Group feedback sessions offer physician groups room for reflection on audit results. During the session, a focus should be placed on building trust in the data and investing in mutual relationships among physician group members, and among the facilitators and physician group members. Special attention to facilitator training, attendance by key group members, and the level of feedback can enhance a group’s ability to discuss actions to improve the quality of care. The facilitator plays an important role in supporting the group in interpreting the results, steering the discussion toward sharing change cues, and helping the physician group in developing action plans. Since (routine care) data is becoming increasingly available, A&F is more appealing to improve quality of care, making it important to perform further research on how to effectively facilitate group feedback sessions. By implementing the lessons learned from this study, group feedback sessions can be refined, supporting participants in action planning.

## Supplementary Information

Additional file 1. Supplementary material.

Additional file 2. Coreq Checklist.

Additional file 3. Example Slides Group Feedback Session.

Additional file 4. Structured questionnaire for facilitators for the evaluation of the group feedback sessions.

## Data Availability

The data supporting the findings of this study are not openly available for reasons of confidentiality and are available from the corresponding author upon reasonable request.

## Abbreviations

A&F: Audit and feedback
LRTI: Lower respiratory tract infection
CRP (POCT): C-reactive protein (point-of-care testing)
