# Supplementary material for: Audit and group feedback in nursing home physician groups: lessons learned from a qualitative study
Source: BMC Health Serv Res. 2025 Feb 11;25:227. doi: 10.1186/s12913-025-12355-y (PMC11817538; doi:10.1186/s12913-025-12355-y)
Supplement: Supplementary file 1 — Additional file 1. Supplementary material. [file 12913_2025_12355_MOESM1_ESM.pdf]

Content Supplementary material

- Supplementary Box 1 Group feedback session outline: Lower respiratory tract infection section
- Supplementary Table 1 Descriptions of the facilitators and research members
- Supplementary Figure 1 Adherence rates for guideline recommendations for lower respiratory tract infections by physician groups

Supplementary Box 1 Group feedback session outline: Lower respiratory tract infection section

1. Introduction to audit and feedback program and antibiotic medication theme	*Introduction*	*3 min*
2. Introduction to lower respiratory tract infection (LRTI) results		
3. Guideline adherence for antibiotic treatment initiation	*Diagnostics*	*15-20 min*
3a. Statement: I know the flowchart from the guideline on LRTIs by heart.		
3b. Adherence rates		
3c. Reasons for non-adherence		
3d. Adherence rate compared to previous cycle (if applicable)		
4. Guideline adherence for C-reactive protein (CRP) test		
4a. Statement: We generally adhere with the guideline when performing the CRP test.		
4b. Percentage of appropriately performed CRP tests		
4c. Percentage of appropriately abstained CRP tests		
5. Guideline adherence for prescribed antibiotics	*Antibiotics*	*5-10 min*
5a. Adherence rates		
5b. Statement: We prescribe the recommended antibiotics more often than last year.		
5c. Adherence rates compared to previous cycle (if applicable)		
6. Overview of antibiotics prescribed		
7. Recap of lower respiratory tract infection results	*Recap*	*2 min*
8. Urinary tract infection results*	*Introduction; Diagnostics; Antibiotics; Recap*	*30 min*
9. Psychotropic medication results (if applicable)*	-	*30 min*

*These topics are not part of this analysis.
Timeline for indicative references (Note: sessions followed a flexible and not a strict schedule).

Supplementary Table 1 Descriptions of the facilitators and research members

**Faculty facilitators**	
FF1	Elderly care physician (f) who still works after retirement as a teacher at the university's medical residency program and as an expert physician (SCEN physician) involved in providing support, advice, and formal consultation to physicians with patients who have requested euthanasia. First year involved as a facilitator.
FF2	Elderly care physician (m) who still works after retirement in practice as an attending locum, combining this with teaching at the university's medical residency program. First year involved as a facilitator.
FF3	Elderly care physician (m) who still works after retirement in practice as an attending locum, has taught at the university's medical residency program, and involved in national and regional antimicrobial stewardship programs for nursing homes. Already involved as facilitator in the group feedback sessions of the first cycle.
**Research facilitators**	
FR1	Post-doctoral researcher (f) with a background in physical therapy and human movement sciences. Involved in the sentinel network since 2021 and attended the feedback sessions in 2022 and 2023. During the 2022 cycle of the sentinel network had regular contact with the physicians of the participating nursing homes for coordination of the project. I was therefore fairly well informed about the functioning of the medical team. In both 2022 and 2023 I was involved in the analyses of the data and preparation of results presented during the feedback sessions. In my role as a researcher during the feedback sessions I was responsible for conveying the results in an objective way, which I believe was not influenced by the fact that I knew more about the performance of some of the medical teams.
FR2 (GY)	PhD student (m), together with CA main researchers of this study. First qualitative study with transcript coding for me. A physician trained as hospitalist who switched to nursing home care, due to the better mix of cure and care compared to the hospital setting. I believe that data can help physicians to deliver high-quality care. Reading about the Calgary Framework for the first time, I could strongly relate to how peers and stakeholders respond to data in the network's group feedback sessions and other previous quality improvement work in which I have been involved. Second year as a facilitator to these group feedback sessions, and the first year almost all sessions were held online due to the COVID pandemic. This year I presented to some groups for the second time and recognized some of the participants, but did not feel that this was mutual. Overall, across all groups, there were at most a handful of participants whom I had previously met as peers during medical training or as a supervising physician. I had the feeling that it was easier to connect in the groups with these pre-existing relationships.

FR3	Elderly care physician and PhD student (f), started working as a researcher in the sentinel network just before the 2023 A&F sessions. In half of the attended sessions, I knew one to three physicians beforehand. This helped me to feel at ease, although in other sessions I also felt fine. Having been working in several physician groups in nursing homes in the past seven years, the dynamics and manners of such groups were familiar to help me understand the difficulties of implementing and de-implementing in nursing home settings. As a peer, I was able to show some compassion with the physicians. On the other hand, I felt it was my task as a researcher to support peers to embrace new knowledge and encourage them to incorporate evidence based medicine in daily practice.
FR4 (PJ)	Postdoc researcher (f), background in nursing (bachelor) and health sciences (master).<br>I was coordinator of the sentinel network in which >20 nursing homes participated.<br>In the half year before the sessions, when the nursing homes collected the data, I contacted the contact person (elderly care physician or nurse specialist) several times to evaluate the data collections, to discuss the number of completed forms, motivate them to formulate specific learning objectives, and to answer any questions. This provided me with insights into the organization and motivation to participate within the sentinel network. This might have influenced my ideas about the way in which these organizations felt open to receive feedback and to improve care. However, it was my first experience with audit and feedback and I tried to be open towards the way in which these sessions would proceed.
**Other research members**	
CA	PhD student (f), together with GY the main researcher of this study. Some experience in qualitative research, but the first time using the framework method. I am trained in health economics and policy, specifically in the long-term care setting, but not as a physician. Because I did not participate in the group feedback sessions, I did not know the participants. Given I am not trained in healthcare practice, I could contribute with a more open view about what happened during the sessions. Besides, I could not compare experiences in current sessions to experiences of last year and I was unable to use any medical knowledge to underpin certain reactions or behavior. This has therefore contributed to a more independent perception and reflection.
MS	Professor of Medicine for Older People (m), with training in medical education, knowledge translation and experience in quantitative as well as qualitative research methods. Research focus on dementia in long-term care, infections in long-term care and postgraduate medical education.<br><br>As a clinician in long term care I participated for several years in pharmacotherapy audit meetings resembling the A&F meetings that were the focus of the current study.

	As former head of the resident training for ECPs at Amsterdam UMC I had contact with some of the organizations in another role. I did not participate in data collection and in the analysis only anonymized written data were used.<br><br>The long-term care sector is characterized by limited staff and high administrative burden with priority given to patient care. Participation in quality improvement and research projects comes next to that.
MdB	Professor of Public Health (f), especially Quality of Care, trained as a public health physician, with a background in epidemiology, medical informatics, and value based health care. Because I did not participate in the group feedback sessions, I did not know the participants, I could contribute with a more open view to what happened during the sessions. My experience with audit and feedback in hospital settings formed my perception and reflection to some extent. It especially made us aware of more generic aspects which were similar in both setting, and many aspects that differ and seemed to be specific due to the characteristics of the medical discipline or the maturity of quality of care practices in a specific setting.
KJ	Assistant professor at the dept of Medicine for Older People (f). Clinical epidemiologist and health scientist with a research focus on dementia caregiving and secondary use of routine care data for research and quality improvement. Project leader of the national program in which that the sentinel network is embedded. I have coordinated the network in the first cycle during this program and had contact with the physicians of the participating nursing homes. I attended some A&F sessions as a researcher in this first cycle. Based on these experiences, I had ideas about how ‘well’ the physician group functioned. As only anonymized written data were used in the analysis, I did not know which nursing home it concerned.

(f = female, m = male)

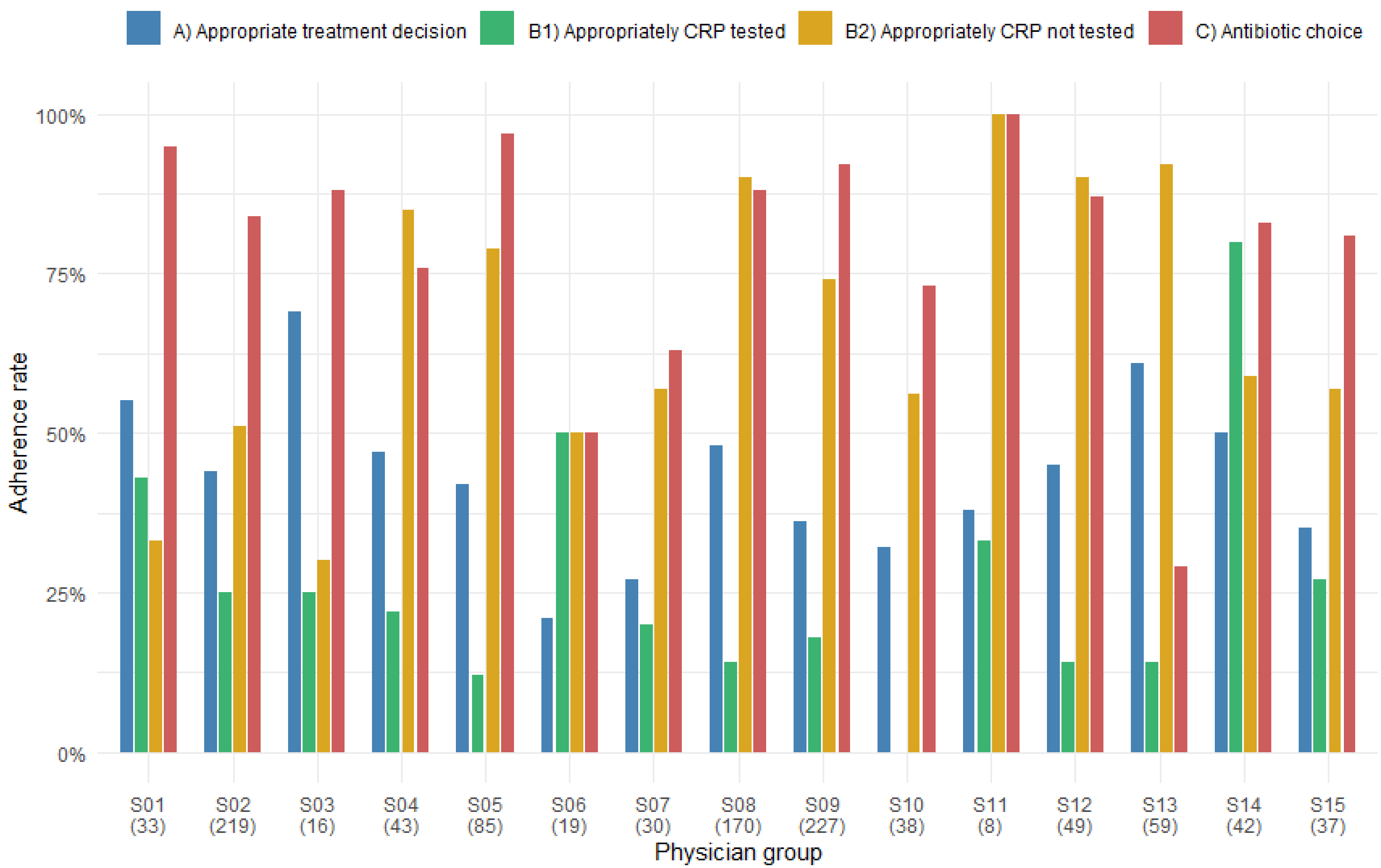

Supplementary Figure 1 Adherence rates for guideline recommendations for lower respiratory tract infections by physician groups (number of recorded audit forms in parentheses)
