# Supplementary material for: Audit and group feedback in nursing home physician groups: lessons learned from a qualitative study
Source: BMC Health Serv Res. 2025 Feb 11;25:227. doi: 10.1186/s12913-025-12355-y (PMC11817538; doi:10.1186/s12913-025-12355-y)
Supplement: Supplementary file 2 — Additional file 2. Coreq Checklist. [file 12913_2025_12355_MOESM2_ESM.pdf]

Consolidated criteria for reporting qualitative studies (COREQ): 32-item checklist

Developed from:
Tong A, Sainsbury P, Craig J. Consolidated criteria for reporting qualitative research (COREQ): a 32-item checklist for interviews and focus groups. *International Journal for Quality in Health Care*. 2007. Volume 19, Number 6: pp. 349 – 357

YOU MUST PROVIDE A RESPONSE FOR ALL ITEMS. ENTER N/A IF NOT APPLICABLE

No. Item	Guide questions/description	Reported on Page #
**Domain 1: Research team and reflexivity**		
Personal Characteristics		
1. Inter viewer/facilitator	Which author/s conducted the inter view or focus group?	Table 1
2. Credentials	What were the researcher's credentials? E.g. PhD, MD	Supplementary Table 1, Additional file 1
3. Occupation	What was their occupation at the time of the study?	Supplementary Table 1, Additional file 1
4. Gender	Was the researcher male or female?	Supplementary Table 1, Additional file 1
5. Experience and training	What experience or training did the researcher have?	Supplementary Table 1, Additional file 1
Relationship with participants		
6. Relationship established	Was a relationship established prior to study commencement?	Supplementary Table 1, Additional file 1
7. Participant knowledge of the interviewer	What did the participants know about the researcher? e.g. personal goals, reasons for doing the research	Methods
8. Interviewer characteristics	What characteristics were reported about the inter viewer/facilitator? e.g. Bias, assumptions, reasons and interests in the research topic	Supplementary Table 1, Additional file 1
**Domain 2: study design**		
Theoretical framework		
9. Methodological orientation and Theory	What methodological orientation was stated to underpin the study? e.g. grounded theory, discourse analysis, ethnography, phenomenology, content analysis	Methods
Participant selection		

10. Sampling	How were participants selected? e.g. purposive, convenience, consecutive, snowball	Methods
11. Method of approach	How were participants approached? e.g. face-to-face, telephone, mail, email	Methods
12. Sample size	How many participants were in the study?	Table 1
13. Non-participation	How many people refused to participate or dropped out? Reasons?	N/A
Setting		
14. Setting of data collection	Where was the data collected? e.g. home, clinic, workplace	Methods
15. Presence of non-participants	Was anyone else present besides the participants and researchers?	Methods
16. Description of sample	What are the important characteristics of the sample? e.g. demographic data, date	Table 1
Data collection		
17. Interview guide	Were questions, prompts, guides provided by the authors? Was it pilot tested?	N/A
18. Repeat interviews	Were repeat inter views carried out? If yes, how many?	N/A
19. Audio/visual recording	Did the research use audio or visual recording to collect the data?	Methods
20. Field notes	Were field notes made during and/or after the inter view or focus group?	Methods
21. Duration	What was the duration of the inter views or focus group?	Methods
22. Data saturation	Was data saturation discussed?	N/A
23. Transcripts returned	Were transcripts returned to participants for comment and/or correction?	N/A
**Domain 3: analysis and findings**		
Data analysis		
24. Number of data coders	How many data coders coded the data?	Methods
25. Description of the coding tree	Did authors provide a description of the coding tree?	Figure 1
26. Derivation of themes	Were themes identified in advance or derived from the data?	Methods
27. Software	What software, if applicable, was used to manage the data?	Methods
28. Participant checking	Did participants provide feedback on the findings?	N/A
Reporting		
29. Quotations presented	Were participant quotations presented to illustrate the themes/findings? Was each quotation identified? e.g. participant number	Results
30. Data and findings consistent	Was there consistency between the data presented and the findings?	Results
31. Clarity of major themes	Were major themes clearly presented in the findings?	Results

32. Clarity of minor themes	Is there a description of diverse cases or discussion of minor themes?	Results/Discussion

Once you have completed this checklist, please save a copy and upload it as part of your submission. When requested to do so as part of the upload process, please select the file type: *Checklist*. You will NOT be able to proceed with submission unless the checklist has been uploaded. Please DO NOT include this checklist as part of the main manuscript document. It must be uploaded as a separate file.
