# Supplementary material for: Audit and group feedback in nursing home physician groups: lessons learned from a qualitative study
Source: BMC Health Serv Res. 2025 Feb 11;25:227. doi: 10.1186/s12913-025-12355-y (PMC11817538; doi:10.1186/s12913-025-12355-y)
Supplement: Supplementary file 3 — Additional file 3. Example Slides Group Feedback Session. [file 12913_2025_12355_MOESM3_ESM.pdf]

Background information

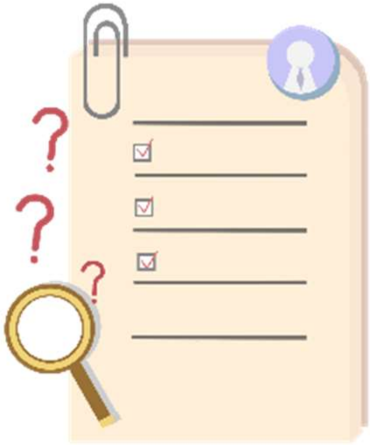

170

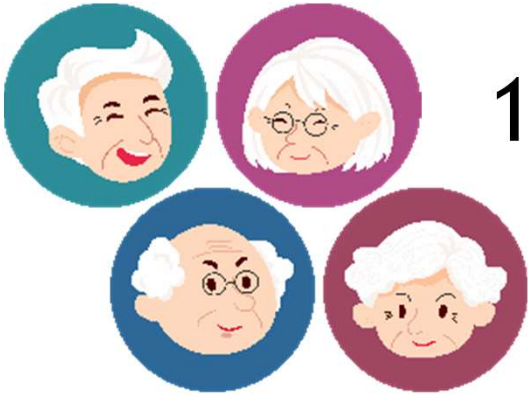

152

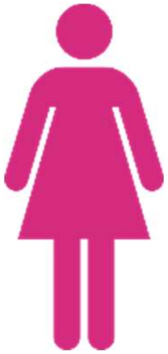

59%

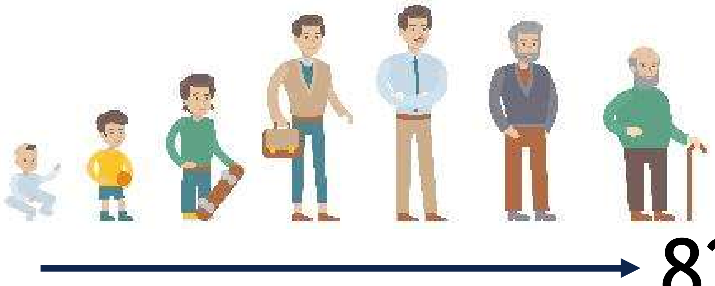

82 years

INTRODUCTORY STATEMENT

I know the flowchart from the guideline on LRTIs by heart

Were the flowcharts followed?

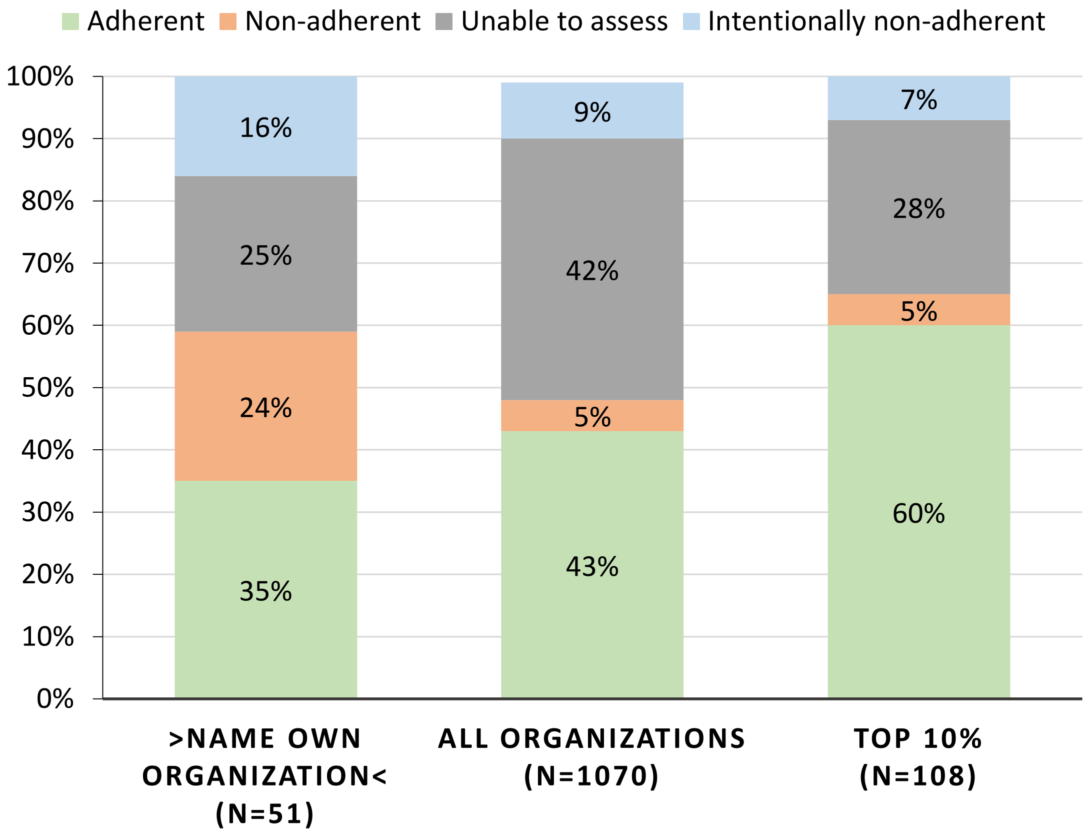

Reasons for non-adherence

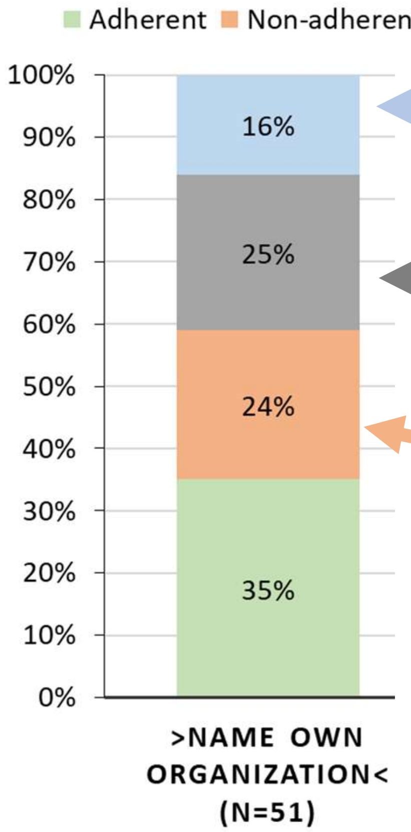

Intentionally non-adherent:

- 1x consultation medical specialist hospital
- 3x treatment preferences
- 4x other

Unable to assess:

- 4x no CRP result
- 3x no cough, no fever, and no delirium
- 2x no information on auscultation and/or tachypnea
- 4x cough, fever, and delirium absent or unknown

Non-adherent:

- 5x no AB initiated while there was an indication
- 7x AB initiated while no indication

Were the flowcharts followed compared to audit (2021-2022)?

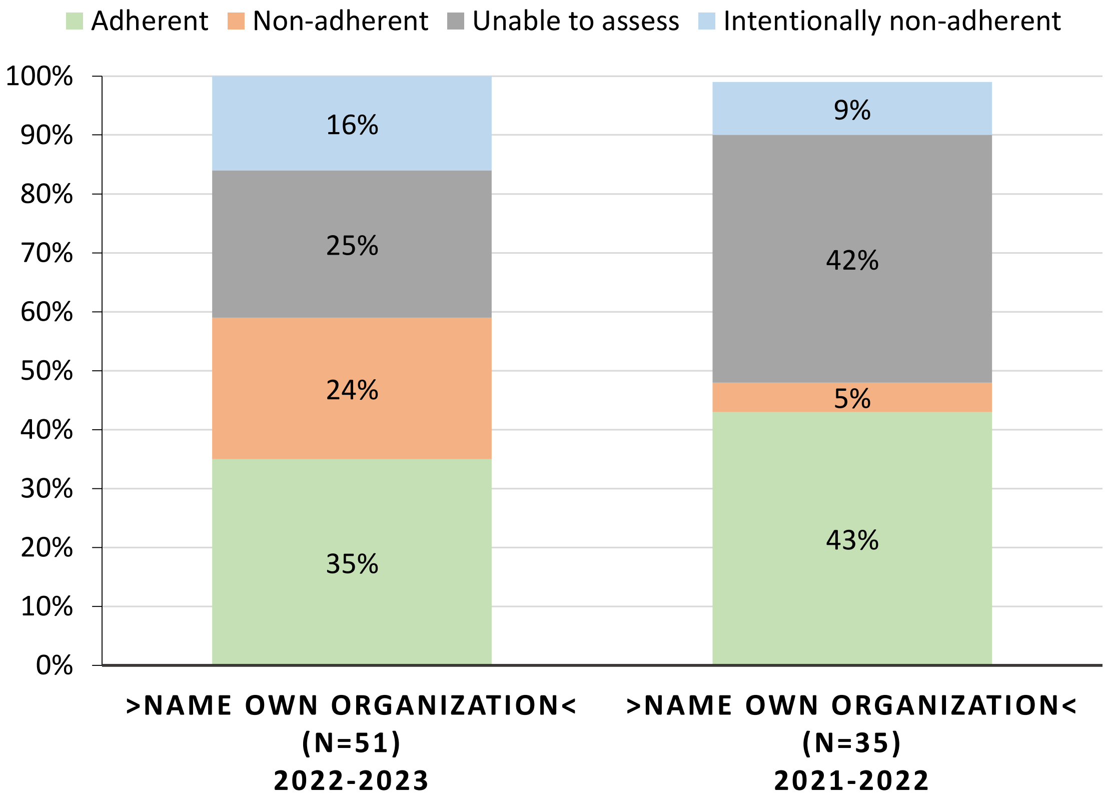

What do you notice?

INTRODUCTORY STATEMENT

We generally adhere to the guideline when performing the CRP test.

Was the CRP test performed in accordance with the flow chart?

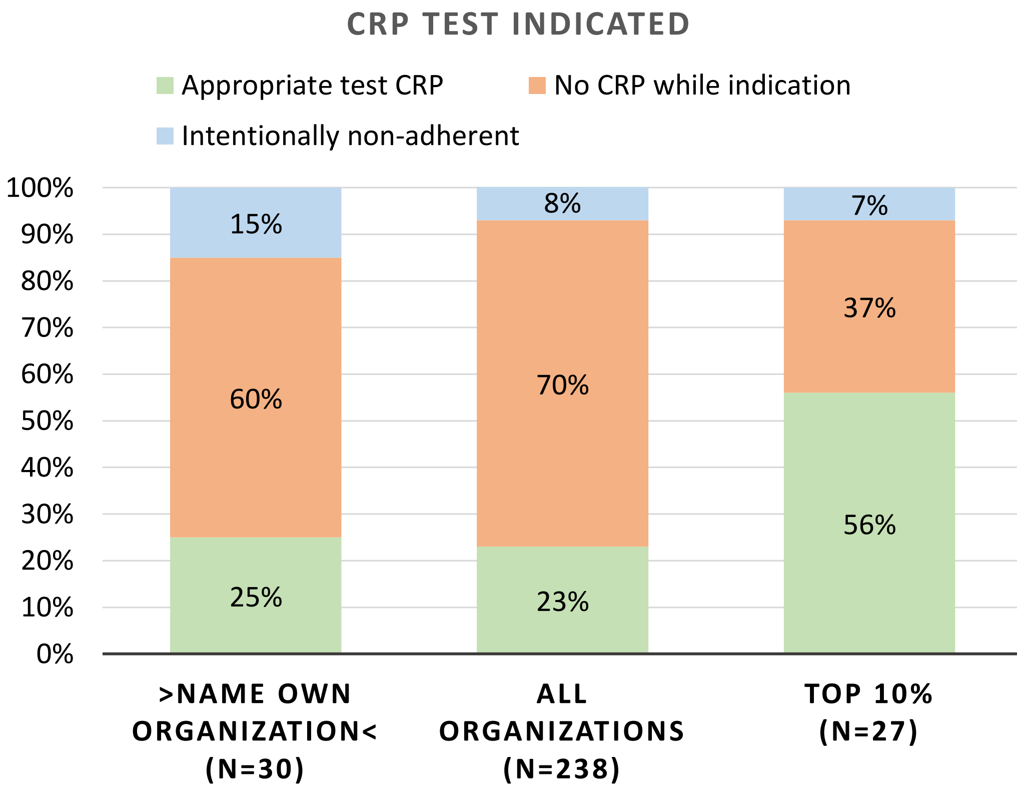

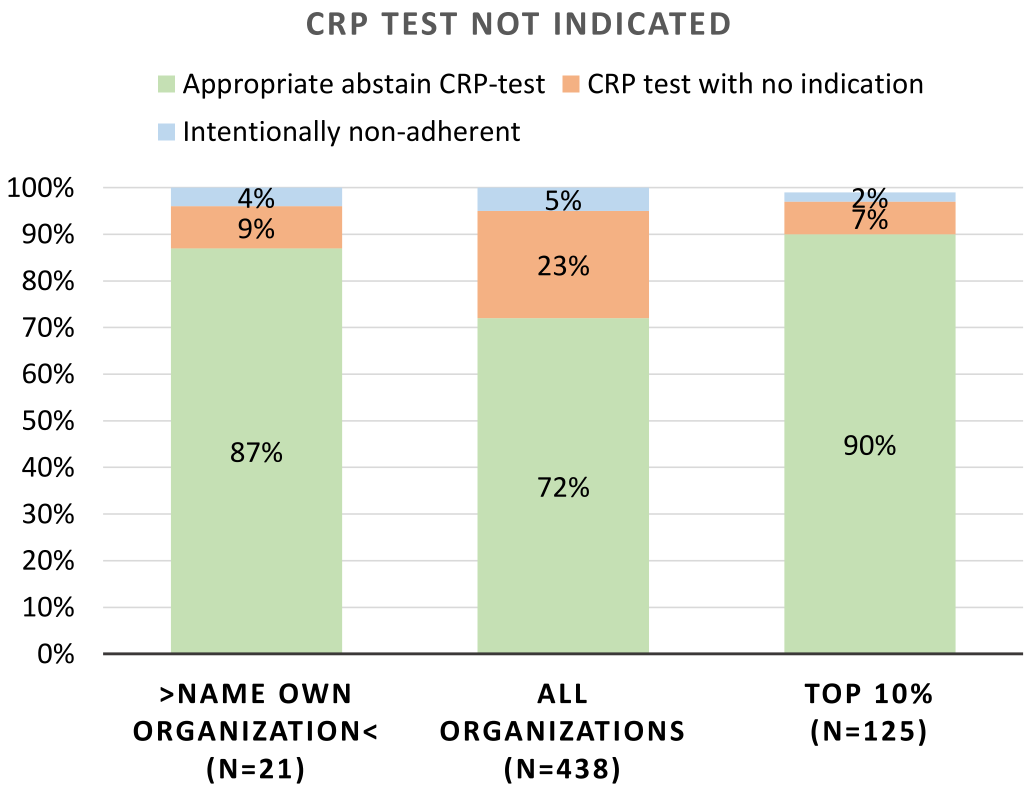

Are the results recognizable?

Is the prescribed AB in adherence with the guideline?

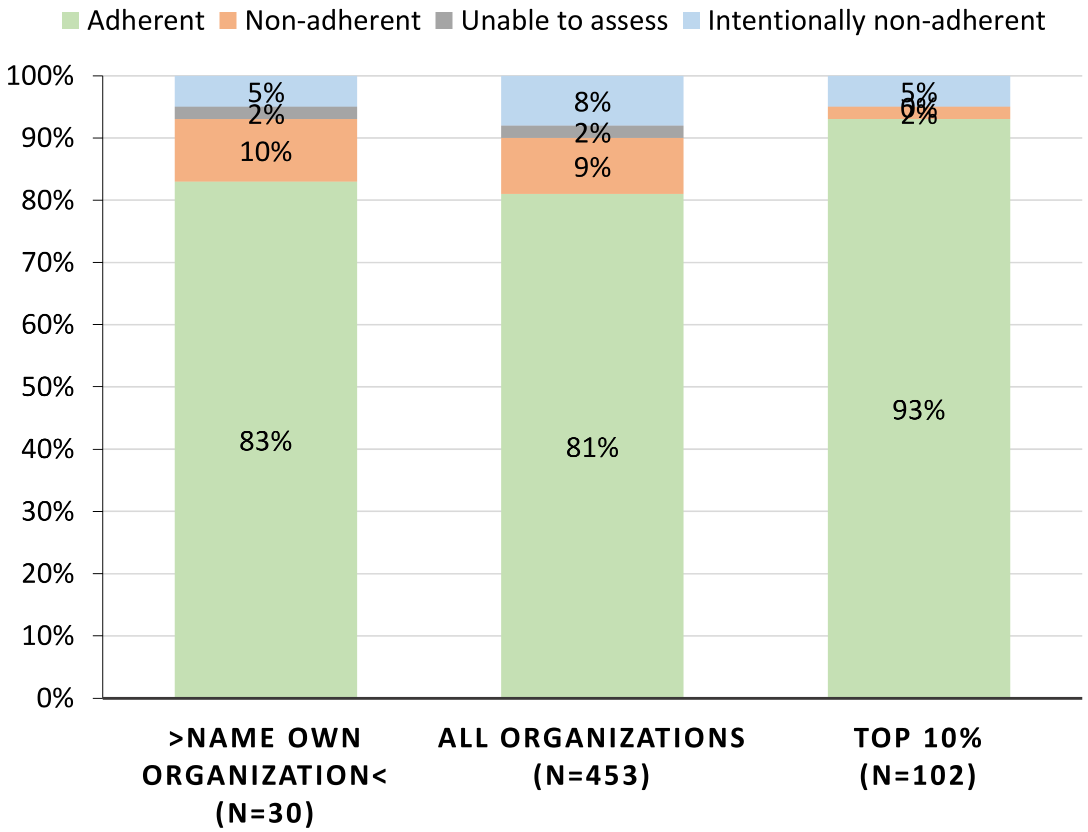

N = total number of forms with AB prescriptions and work diagnosis LRTI

INTRODUCTORY STATEMENT

We prescribe the recommended antibiotics more often than last year

Prescribed AB compared to last audit (2021-2022)

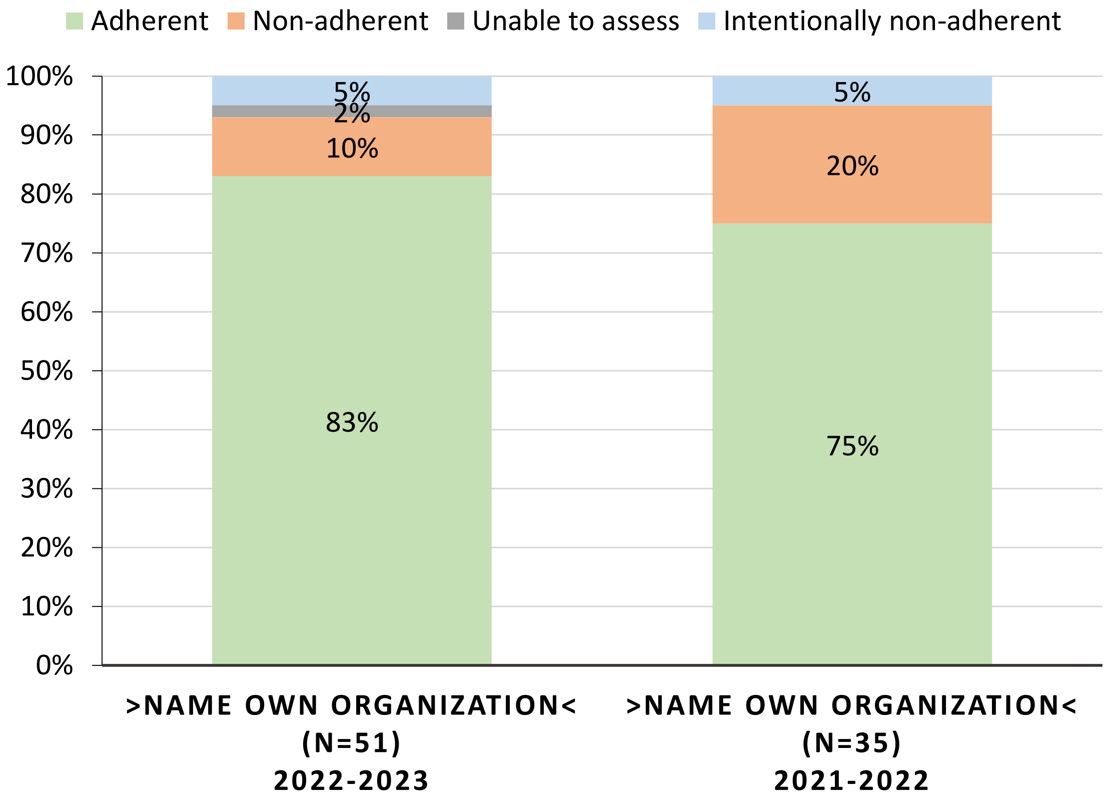

Types of AB prescribed

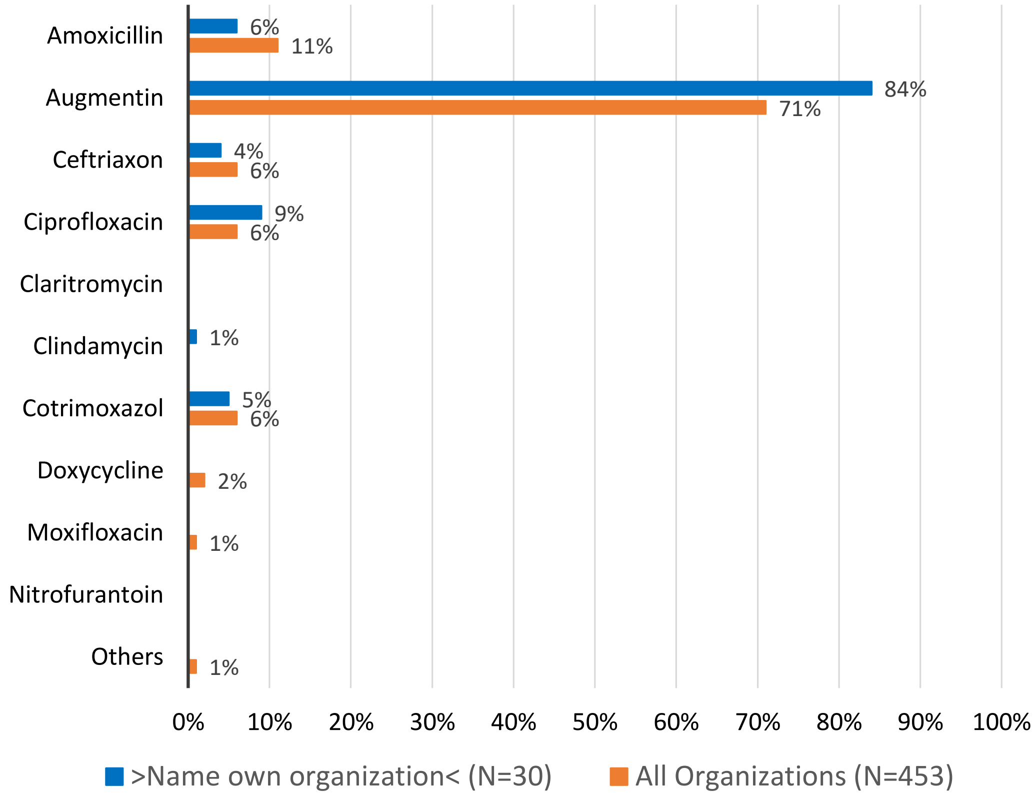

N = total number of forms with AB prescriptions and work diagnosis LRTI

Types of AB prescribed compared to last audit (2021-2022)

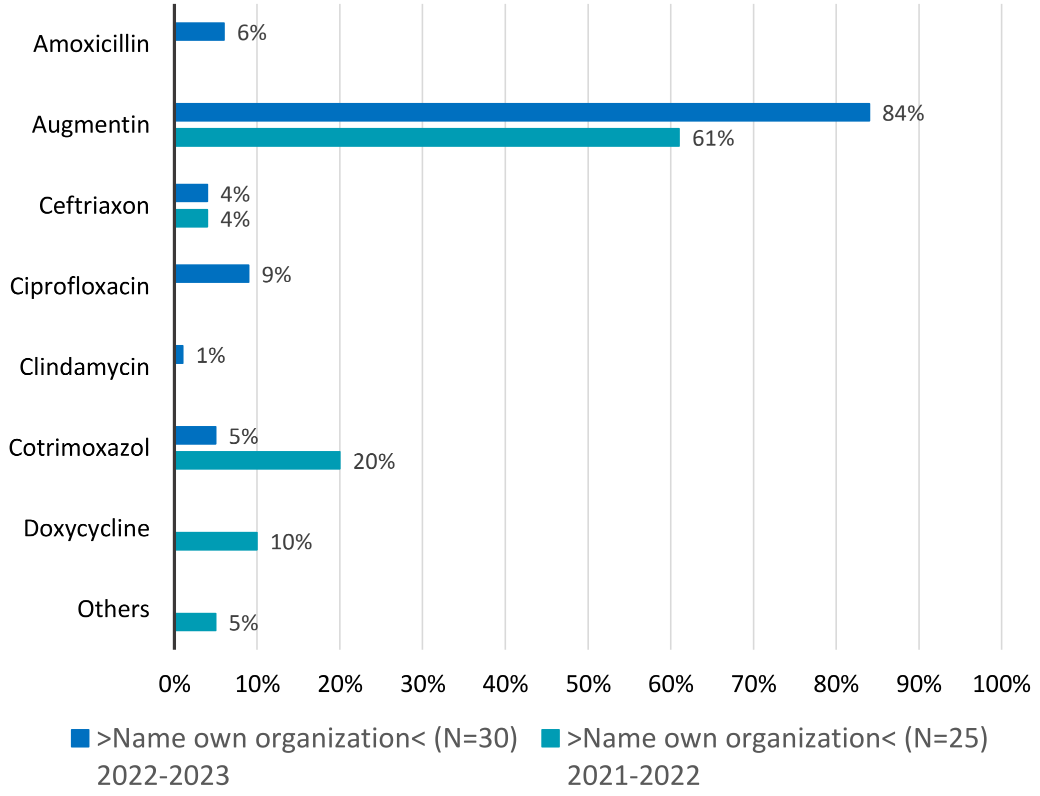

Is this as you expected?

What is in the local formulary?

Recap results LRTI

	What went well?	What can be improved?
Diagnostics		
Treatment		
