# Supplementary material for: Audit and group feedback in nursing home physician groups: lessons learned from a qualitative study
Source: BMC Health Serv Res. 2025 Feb 11;25:227. doi: 10.1186/s12913-025-12355-y (PMC11817538; doi:10.1186/s12913-025-12355-y)
Supplement: Supplementary file 4 — Additional file 4. Structured questionnaire for facilitators for the evaluation of the group feedback sessions. [file 12913_2025_12355_MOESM4_ESM.pdf]

Additional file 4 Structured questionnaire for facilitators for the evaluation of the group feedback sessions

The evaluation questionnaire used in this study is based on a questionnaire developed for general practitioners in a similar A&F program in the Netherlands, “Spiegelaar” (for more information about the program in Dutch, please visit: www.amsterdamsehuisartsen.nl/thema/kwaliteit/)

Introduction

With this questionnaire, we evaluate how you perceived the group feedback sessions within the Nursing home network.

We will use the results of this questionnaire for scientific research to further improve the A&F information. You can read more about the study in the Information Letter.

Completing the questionnaire takes approximately five minutes.

By completing the questionnaire, you consent to the use of your data in the study, as mentioned below.

Click Next to start the questionnaire.

Consent for study ‘Evaluation of A&F information within nursing home network of “Leren van Data”.
- By completing the questionnaire, you consent to the collection and use of your data in the manner and for the purposes specified in the Information Letter.
- You have read the Information Letter. You were also able to ask questions. Your questions have been answered sufficiently. You had enough time to decide whether to participate.
- You know that participating is voluntary. You also know that you can decide to stop completing the questionnaire at any time. You do not have to provide a reason for doing so.
- By completing the questionnaire, you give permission for your completed data to be stored within the Amsterdam UMC, location VUmc, for another 10 years after this study.

Start questionnaire

1: Which group feedback session did you just attend?

>*Answer options list of names of participating nursing home organization*<

2: What was your role in this group feedback session?

o Faculty facilitator
o Research facilitator
o Observer

2b: If “Research facilitator” was chosen, the following questions were asked:

How many participants were present in person during the group feedback session? [Number between 0-90]
How many participants were present online during the group feedback session? [Number between 0-90]

How many of the participants were elderly care physicians? [Number between 0-90]
How many of the participants were medical residents in training for elderly care medicine? [Number between 0-90]
How many of the participants were medical residents in training for general practice medicine? [Number between 0-90]
How many of the participants were nurse practitioners? Including those in training. [Number between 0-90]
How many of the participants were physician assistants? Including those in training. [Number between 0-90]
How many of the participants were junior doctors? [Number between 0-90]
Other, if applicable? Function and number of participants [open text field]

3: To what extent do you agree with the following statements:
0 = completely disagree; 9 = completely agree.

During the group feedback session …

	Completely disagree 0	1	2	3	4	5	6	7	8	Completely agree 9	Not applicable
The participants were inspired	○	○	○	○	○	○	○	○	○	○	○
The participants asked us questions	○	○	○	○	○	○	○	○	○	○	○
The participants asked each other questions	○	○	○	○	○	○	○	○	○	○	○
The participants brought up points for discussion	○	○	○	○	○	○	○	○	○	○	○
The participants felt safe to discuss each other's practices	○	○	○	○	○	○	○	○	○	○	○
There was interaction between the participants	○	○	○	○	○	○	○	○	○	○	○
There was enough time to discuss everything	○	○	○	○	○	○	○	○	○	○	○

Participants felt that the A&F results gave a representative picture of the performance of the physician group	○	○	○	○	○	○	○	○	○	○	○
Participants gained new insights into this topic	○	○	○	○	○	○	○	○	○	○	○
Participants set specific goals for improvement	○	○	○	○	○	○	○	○	○	○	○
I expected participants to succeed in making improvements	○	○	○	○	○	○	○	○	○	○	○

4: Following the A&F results
What is going well in this organization?
[open text field]

5: According to the A&F results
What can be improved in this organization?
[open text field]

6: Following this group feedback session
What has this organization agreed on as action plans?
[open text field]

7: What rating do you give this group feedback session?
(rating: 1 poor – 10 excellent)
[Number between 1-10]

8: What do you think went well during this group feedback session?
[open text field]

9: How do you think this group feedback session could have been improved?
[open text field]

10: Room for comments/remarks
[open text field]

Thank you very much for your participation. Your answers have been sent.
